# Virtual screening, Docking, ADMET and System Pharmacology studies on Garcinia caged Xanthone derivatives for Anticancer activity

**DOI:** 10.1038/s41598-018-23768-7

**Published:** 2018-04-03

**Authors:** Sarfaraz Alam, Feroz Khan

**Affiliations:** 1 Metabolic & Structural Biology Department, CSIR-Central Institute of Medicinal & Aromatic Plants, P.O.-CIMAP, Lucknow, 226015 Uttar Pradesh India; 2 Academy of Scientific & Innovative Research (AcSIR), CSIR-CIMAP Campus, Lucknow, 226015 Uttar Pradesh India; 3 Present Address: Skaggs School of Pharmacy & Pharmaceutical Sciences, University of California San Diego (UCSD), 9500 Gilman Drive, La Jolla, San Diego, CA 92093 USA

## Abstract

Caged xanthones are bioactive compounds mainly derived from the *Garcinia* genus. In this study, a structure-activity relationship (SAR) of caged xanthones and their derivatives for anticancer activity against different cancer cell lines such as A549, HepG2 and U251 were developed through quantitative (Q)-SAR modeling approach. The regression coefficient (r^2^), internal cross-validation regression coefficient (q^2^) and external cross-validation regression coefficient (pred_r^2^) of derived QSAR models were 0.87, 0.81 and 0.82, for A549, whereas, 0.87, 0.84 and 0.90, for HepG2, and 0.86, 0.83 and 0.83, for U251 respectively. These models were used to design and screened the potential caged xanthone derivatives. Further, the compounds were filtered through the rule of five, ADMET-risk and synthetic accessibility. Filtered compounds were then docked to identify the possible target binding pocket, to obtain a set of aligned ligand poses and to prioritize the predicted active compounds. The scrutinized compounds, as well as their metabolites, were evaluated for different pharmacokinetics parameters such as absorption, distribution, metabolism, excretion, and toxicity. Finally, the top hit compound 1G was analyzed by system pharmacology approaches such as gene ontology, metabolic networks, process networks, drug target network, signaling pathway maps as well as identification of off-target proteins that may cause adverse reactions.

## Introduction

Cancer is a major public health problem worldwide and is the second-leading cause of death in the United States. In 2016, projected new cancer cases and cancer deaths in the USA are 1,685,210 and 595,690 respectively^1^. The burden of cancer will increase to 23.6 million new cases by 2030^2^. The Indian Council of Medical Research has estimated that about 14.5 lakh patients will develop cancer in India during 2016 with the number expected to rise to 17.3 lakh by 2020^3^. This high mortality rate throughout the world, make the pharmaceutical companies as well as the scientific community to have an acquisitive appetite for new lead identification. One of the important sources of drug discovery is natural products. Over the past 30 years, more than 50% of the drugs developed in the pharmaceutical industry are natural products or inspired by their structure^4^. In the case of anticancer drugs, the percentage is even higher. The Camptothecin (*Camptotheca acuminate)*, Vinblastine & Vincristine (*Catharanthus roseus*), Paclitaxel (*Taxus brevifolia*), Podophyllotoxin (*Podophyllum peltatum*) and its semi-synthetic derivatives known as Etoposide and Teniposide^5^ etc. is its best example. One such promising tree is Garcinia, a plant genus of the family Clusiaceae, native to Asia, Australia, tropical and southern Africa. The best-known species in India are *G. mangostana*, *G. gummi-gutta*, and *G. hanburyi*. The gambogin resin secreted by the Garcinia genus of tropical plants has been used as folk medicine for centuries in Southeast Asia^6^. The major bioactive constituents of the gamboges resin secreted primarily by *G. hanburyi*^7^. Studies with this resin extract led to the identification of a new class of natural products that are collectively referred to as caged *Garcinia* xanthones based on their unique 4-oxa-tricyclo [4.3.1.0^3,7^] dec-2-one caged scaffold with a common xanthone backbone^8^.

Prior studies suggest that Garcinia xanthone and its derivatives have significant *in vitro* anticancer activity with promising pharmacology, but the molecular reason behind the activity is not yet explored. Keeping in mind the unusual caged skeleton and remarkable bioactivity of Garcinia xanthones, the present work reports the identification of pharmacophore features and activity controlling sites, along with the identification of the mechanism of action based on the structure-activity relationship which led to virtual screening of a caged xanthone derivatives library, for the identification of anticancer lead compounds. These are achieved by using the combined approach of Quantitative Structure-Activity Relationship (QSAR), docking, Absorption, Distribution, Metabolism & Toxicity (ADMET) and system pharmacology in a pipeline^9–12^. The QSAR permits the quantification of the relation between structure (described by selected properties or descriptors) of the ligand and its biological activity. It also helps in the optimization of the groups that modulate the potency as well as in the rationalization of the compound which leads to better activities and can also be used as a screening tool^13^. The efficacy of a drug also depends on its binding mode and affinity toward the target site, somehow the docking study help to perceive this efficacy and affinity^14^.

In this work three QSAR models were developed for anticancer activity as per OECD (Organization for Economic Co-operation and Development) regulatory purposes guidelines such as (i) a defined endpoint (ii) an unambiguous algorithm; (iii) a defined domain of applicability; (iv) appropriate measures of goodness-of-fit, robustness and predictive power and (v) a mechanistic interpretation^15^ and validated through different statistical parameters. These models were developed by adopting multiple linear regression (MLR) method using the training data set of diverse but *in vitro* experimentally known cytotoxic/anticancer activity of caged xanthones and its derivatives against human cell lines, namely alveolar adenocarcinoma cell line (A549), liver hepatocellular carcinoma cell line (HepG2), and glioblastoma cell line (U251). The derived QSAR models quantified the chemical descriptors or properties and predicted the inhibitory concentration to 50% of the population (IC_50_) of each designed compound thereby highlighting its range of clinical efficacy and toxicity. The derived chemical properties were used to design 1000 compounds and advanced to screen through the QSAR models. Those compounds having the IC_50_ value of more than 15 µM was excluded.

Further, 25 designed compounds were filtered through Lipinski’s rule of five, along with ADMET risk parameters assessments. In addition to optimizing the screening and testing by looking at only the most promising compounds for its early information on ADMET data so to reduce the risk of late-stage attrition. The parameters of the risk were also provided so that it should be removed when designing the drugs. Later an appraisal of synthetic accessibility of compounds was performed, which gives an idea to the easiness of synthetic possibility^16^. Prior to docking studies, the target identification work is carried out. With the receptor model identification, in next the ligands were identified with the help of molecular docking studies. After manually scrutinizing the top-ranked compounds for novelty, biological potential activity, synthetic accessibility, and through passing pharmacological activity, the compound 1G (IC_50_ of 2.04 µM) was projected for detailed pharmacological studies. Further, this compound and its metabolites were evaluated with system pharmacology aspect. Through this, we identified and ranked cellular pathways and processes most influenced by the compounds 1G by enrichment analysis (EA) i.e. gene ontology (GO) process, metabolic networks, process networks and their pathway maps. These studied were helping to identify the processes, which were up-regulated or down-regulated by the candidate compound as well as to derive a relationship between the effects of the lead compound and biological processes^17^.

Most drugs which affect and metabolize the Cytochrome P450 (CYP450) also affect and hydroxylate various endogenous and xenobiotics compounds such as steroids, cholesterols, lipids, vitamins, or bile salts. It was found through our *in silico* analysis that the compound 1G interface with the Estradiol and Estriol metabolism. Here we study the associated xenobiotic-sensing nuclear receptors, which play key roles in maintaining hepatic cholesterol, steroid, and bile acid homeostasis by interacting with a number of other nuclear receptors and transcription factors. Besides renal excretion and hepatic metabolism, the biliary excretion is the major pathways involved in the removal of xenobiotics. Bile acids facilitate the intestinal digestion and absorption of drugs. This excretion may sometimes consider as phase III metabolism. The bile acid is an important signaling molecule involved in the drug metabolism. The regulatory function of bile acids is mainly a result of bile acid activation of three main intracellular ligand-activated nuclear receptors, such as the farnesoid X receptor (FXR), pregnane X receptor (PXR), and vitamin D receptor. Activation of xenobiotic nuclear receptors by drugs, bile acids, and xenobiotics induces a network of genes involved in phase I, phase II, and phase III drug and bile acid metabolism, transport and detoxification. Thus, we here study the bile acid regulation of lipid metabolism and negative FXR-dependent regulation of bile acid concentration. The uptake of xenobiotics by bile acid transportation system and its regulation, along with the regulation of drug metabolism through neurophysiological process circadian rhythm was also studied^18–20^. The circadian regulation is involved not only in the drug-metabolizing process but also in the elimination of the metabolized compounds through the Hepatobiliary system and the kidney. This system has a strong impact on the pharmacokinetics of drugs and, with the known chromodynamics mechanisms influencing drug efficiency and detoxifications^21^. This effect was demonstrated through a canonical pathway enlisting the enzymes, transporter, transporter ligands, and transcriptional factors involved.

The possible mechanism of action was identified for the candidate compound along with signaling pathway and associated functional genes through the available omics data. However, drugs may also bind to off-target proteins, which affects other biological processes and causes adverse reactions^22^. Most side effects are harmful to humans, but side effects can also be utilized to find new uses for the compounds or the synonyms effect of the compound^23^. We identified such a target which is associated with our lead compound. For the first time, this research article on Garcinia plant derived xanthones, leading to the identification of major anticancer drug targets and therefore defining the mechanism of action. Such study would further establish the development of pharmacophore for drug designing and identification of potential leads against cancer, a disease affecting millions of lives worldwide.

## Methods and Computational Details

### Raw data

Initially a total of 111, 100, and 110 active compounds with reported anticancer activity in terms of (IC_50_; μM) were selected in the training data set for the development of QSAR models correlating structural properties with anticancer activity against three different human cancer cell lines, namely A549, HepG2, and U251 respectively (Tables S1–S6).

### Design and implementation

This section describes the material and methods used for (1) compound standardization, (2) descriptor calculations, (3) QSAR model development, (4) statistical evaluation of the model, and (5) Designing, bioactivity and property prediction for new compounds.

### Compound standardization

The VLifeMDS^®^ software was used to draw the structures, energy calculation and optimization of structural geometries of compounds. The two-dimensional (2D) structures were transformed into three-dimensional (3D) structures by using the converter module of VLife module. The 3D structures were then subjected to energy minimization, which was performed in two steps. The first step was energy minimization using molecular mechanics-2 (MM2) until the root mean square (RMS) gradient value became smaller than 0.1 kcal/mol Å and in the second step, minimized MM2 (dynamics) compounds were subjected to re-optimization through the MOPAC (Molecular Orbital Package) method until the RMS gradient attained a value smaller than 0.0001 kcal/mol Å^24^.

### Descriptor calculation

The descriptors were calculated by using the QSARPlus module of VLifeMDS^®^. These descriptors were grouped into three classes, namely physicochemical descriptors, alignment independent descriptors and atom-type count descriptors. Further, these major classes were categorized in several sub-classes and each individual descriptor has been calculated for each of the training set compounds. In order to select the best subset of descriptors, highly correlated descriptors were excluded. The correlation matrix for derived models was provided in Tables S7–S9.

### QSAR model development

Prior to the QSAR model development selected experimental dataset was divided into the training (Tables S1–S3) and test set (Tables S4–S6). To divide the dataset the sphere exclusion clustering method was used^25^. The validity of training and test set were compared through the UniColoumn statistics approach. Finally, the QSAR models were developed based on stepwise forward MLR method^26^.

### Statistical evaluation/validation of model

Statistical validation is a very important process of robust QSAR model development. Therefore, various types of statistical validations were procured and the best model was selected by applying these different statistical parameters. Listed below are validations performed for the tested alignment, e.g., (i) Coefficient of determination (r^2^) should >0.7, (ii) Leave-one-out cross-validation (LOOcv) or correlation coefficient (q^2^) should be >0.5, (iii) Correlation coefficient of external validation set (pred_r^2^) should be >0.5. This is required to verify the ability of derived model in order to predict the biological activity of similar molecules, (iv) Correlation metrics (rm^2^) calculated based on the correlation between the observed and predicted response data, with or without the intercept and also by interchanging the axes. For an acceptable QSAR model, the value of ‘Average rm^2^’ should be >0.5 & ‘Delta rm^2^’ should be <0.2^24^, (v) Degree of freedom (Df) should be higher (higher is better), (vi) F-test for statistical significance of the model (higher is better, for the same set of descriptors and compounds), (vii) Highest q^2^ value in the randomization test (best_ran_q^2^) should be low as compared to q^2^, (viii) Highest r^2^ value in the randomization test (best_ran_r^2^) should be low as compared to r^2^, (ix) Statistical significance parameter by randomization (alpha_test) should be <0.01, (x) Standard error of estimate (r^2^_se, q^2^_se and, pred_r^2^ se) should be smaller (smaller is better), (xi) Z-score calculated by the randomization test (ZScore) should be higher (higher is better) and, (xii) Applicability Domain (AD)/Extrapolation parameter defined as ‘0’ and ‘1’. The ‘0’ indicate applicability and data are true, whereas ‘1’ indicate the outlier^26^.

#### Designing of novel caged xanthone derivatives

Based on structural feature selection in terms of chemical descriptors and 3D structural components (pharmacophores) of derived QSAR models, activity controlling sites were identified for each model and accordingly novel caged xanthone derivatives were virtually designed.

#### Rule of five, ADMET risk screening and Synthetic accessibility assessment

Lipinski’s rule of five was used for the screening of xanthone derivatives and filtered the orally bioavailable compounds^27^. Further, these filtered xanthone derivatives were evaluated for ADMET Risk parameters by using ADMET Predictor^TM^ (*Simulations Plus*, USA). The overall risk was considered to be in the range of 0–24, where a lower score is preferable and show better druggability^28^. Later the synthetic accessibility was measured in terms of a score on a scale from 1 (very easy to synthesize) to 10 (complex and challenging to synthesize) by using the SYLVIA-XT 1.4^16^.

#### Target identification and Molecular Docking Studies

Target identification: The possible drug targets for the identified hits were explored with the help of MetaDrug^TM^ (Thomson Reuters, USA). Through this, a list of targets is identified, such as ESR1 (Estrogen Receptor 1), GCR (glucocorticoid receptor), FGF (Fibroblast growth factors), CREBP1 (Cyclic AMP Response Element-Binding Protein) and STATs (signal transducer and activator of transcription) (Fig. S1).

### Protein preparation

To prepare the target protein, structure of identifying proteins were retrieved from the RCSB PDB database^29^. In the first step, the protein preparation protocol was used. This protocol performs tasks such as modeling missing loop regions, inserting missing atoms in incomplete residues, deleting alternate conformations and standardizing names of the atoms, protonating titratable residues, and removing water^30^.

### Protein-ligand Docking Studies

The molecular docking and visualization studies were performed with the help of the LibDock program in Discovery Studio v3. 5 (Accelrys, USA)^31^. The LibDock is a flexible docking module. LibDock uses protein site features, referred to as hot spots, consisting of two types states (polar and apolar). The ligand poses are placed into the polar and apolar receptor interactions site. A polar hotspot is preferred by a polar ligand atom (e.g., a hydrogen bond donor or acceptor), and an apolar hotspot is preferred by an apolar atom (e.g., a carbon atom)^32^. The protocol allows the user to specify several modes for generating ligand conformations for docking. Conformer Algorithm based on Energy Screening And Recursive buildup (CAESAR) was used for generating the conformations^33^. The smart minimizer was used for *in situ* ligand minimization. Further, to identify definite interacting residues of the receptor with bound ligand a 2D diagram of docking was also performed. The different scoring protocol was used for the scoring functions such as Jain, Ludi, potential of mean force (-PMF) and piecewise linear potential (PLP1) to evaluate ligand binding in a receptor cavity^34^.

#### In silico pharmacokinetics, metabolism, and toxicity studies

Different pharmacokinetics parameters, namely, Absorption, Distribution, Metabolism, Excretion, and Toxicity were calculated. This study includes the quantitative measurement of drug-like properties, pKa, absorption, solubility, lipophilicity, bioavailability, permeability, volume of distribution, blood-brain-barrier (BBB) penetration, hepatic clearance, transporters, dermal and ocular penetration, plasma-protein binding, metabolism, drug-drug interaction, half-life, etc. A plethora of diverse enzyme families and proteins are involved in xenobiotic metabolism, including CYP450 enzymes. These enzymes were detected for predicted active xanthone derivatives. The different metabolites and the sites of metabolism for Phase I and Phase II metabolism were also calculated. The safety of the compounds is an important parameter for a successful drug. For this, the hepatotoxicity, nephrotoxicity, neurotoxicity, and chance of causing anemia etc. is calculated. We also study the effect of the compound on different liver associated enzymes such as alkaline phosphatase (ALP), gamma-glutamyl transpeptidase (GGT), serum glutamic oxaloacetic (SGOT) & serum glutamate-pyruvate transaminase (SGPT), and lactate dehydrogenase (LDH) enzymes when administered. These findings will be helpful to set dose-ranges. These studies were performed by using the ADMET Predictor^TM^, MetaDrug^TM^, MetaPrint2D, MedChem Designer^TM^ and TOPKAT module of Discovery Studio molecular modeling software^35^.

#### System pharmacology study for enrichment analysis

Identification and ranking of cellular pathways and biological processes which was most influenced by candidate compound 1G were performed by MetaDrug^TM^ tool. For this, the enrichment analysis, such as process networks, metabolic networks, and their pathway map analysis was projected. The enrichment significance was measured in terms of −log (p-value). Lower −log (p-value) means higher the relevance of an entity^26^.

### Signal transduction pathway exploration to identify the possible mechanism of action

In a quest to understand the possible mechanism of action of predicted top hit compound 1G, signal transduction pathway exploration study was performed and identified the pathway and the associated functional genes affected by the top hit compound through MetaDrug^TM^ software.

### Off-targets prediction for compound 1G

Numerous drugs are known for their multi-targeting activities. Thus, keeping in mind these off-target interactions, the complexity of candidate compounds for any such interference with other human protein targets was evaluated by different prediction modules of MetaDrug^TM^ platform.

## Results and Discussion

### QSAR model development and its validation

**Figure 1:**
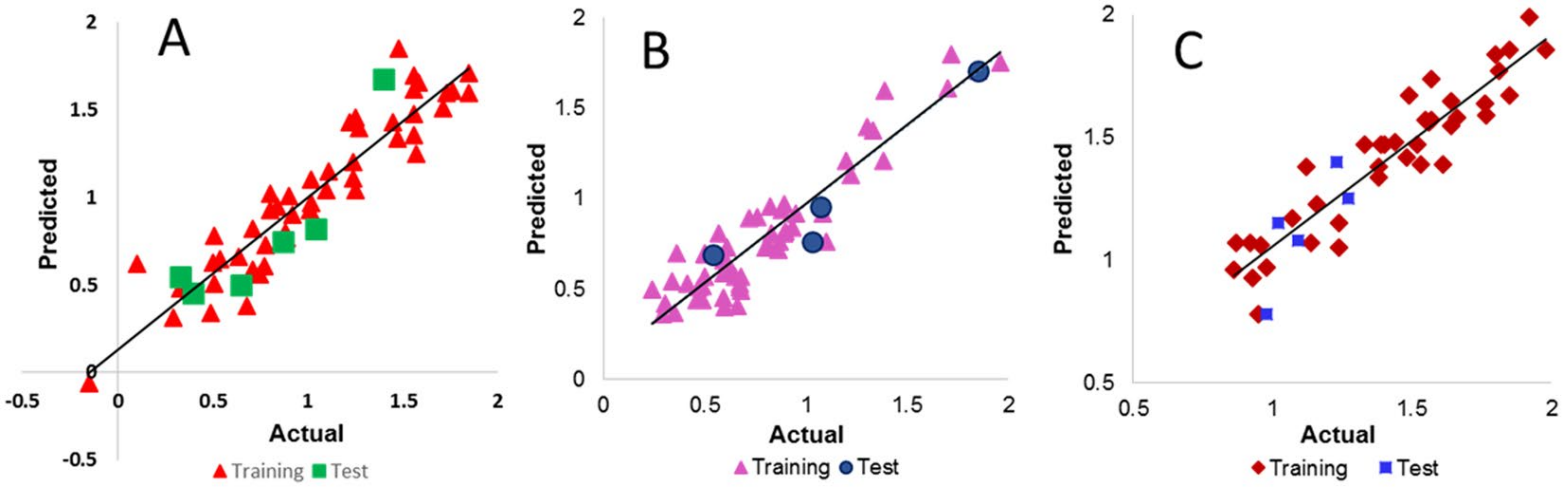
Regression plot representing training and testing of QSAR models. (**A**) Model 1 (**B**) Model 2 (**C**) Model 3.

In the present study, three statistical regression QSAR models were developed, so that to study the xanthones derivatives activity against three different cancer cell lines A549 (Model 1), HepG2 (Model 2), and U251 (Model 3). The study results, several models and the best model was selected based on various statistical parameters such as a square of the correlation coefficient or regression coefficient (r^2^), and the robustness of model predictions was estimated from the cross-validated squared correlation coefficient (q^2^). The plot of observed verses predicted activity provides an idea about how well the model was trained and how well it predicts the activity of the external test set Fig. 1A–C. These derived QSAR models are discussed below in details, and showing the relationship between *in vitro* experimental activity (*i.e*., IC_50_) as the dependent variable and independent variables (chemical descriptors).

#### QSAR Model 1

Result shows that the model 1 yielded a high activity–descriptors relationship accuracy of 87% referred by regression coefficient (r^2^ = 0.87). It showed the internal (q^2^) and external (pred_r^2^) predictive ability of about 81% and 82% respectively. The fitness plot for the training and test set was provided in Fig. 1A. A radar plot representing the closeness between the actual and predicted activity of training and test set compounds was also summarized in Fig. S2. The equation 1 shows that the descriptors which play an important role in determining the anti-cancer activity are SdsCHE-index, MMFF_29, SssssCcount, DeltaEpsilonC; and T_2_2_1. The contributions (positive and negative) aggregate of each of the descriptors was provided in Fig. S3A.

1 $$\begin{array}{rcl}{\bf{Predicted}}\,{\bf{L}}{\bf{o}}{\bf{g}}\,{\bf{I}}{{\bf{C}}}_{{\bf{50}}}({\boldsymbol{\mu }}{\bf{M}}) & = & -39.0090\times {\rm{DeltaEpsilonC}}\\  &  & -0.8078\times {\rm{MMFF}}\_29\\  &  & -1.0827\times {\rm{SssssCcount}}\\  &  & -0.0483\times {\rm{T}}\_2\_2\_1\\  &  & +0.5053\times {\rm{SdsCHE}}-{\rm{index}}\\  &  & -0.0560\end{array}$$

Where,

**DeltaEpsilonC:** A measure of the contribution of electronegativity.

**MMFF_29**: Atom type count descriptor class.SssssCcount: this descriptor defines the total number of carbons connected with four single bonds.

**T_2_2_1:** Count of a number of double-bonded atoms.

**SdsCHE-index**: Electrotopological state indices for a number of –CH group connected with one double and one single bond.

#### QSAR Model 2

This model conceded an activity–descriptors relationship accuracy of 87% referred by regression coefficient (r^2^ = 0.87). The internal (q^2^) and external (pred_r^2^) predictive ability was 84% and 90% respectively. The fitness plot for the training and test set was provided in Fig. 1B. With this, a radar plot which represents the closeness between the actual and predicted activity of the compounds of training and test set were provided in Fig. S4. This model in equation 2 showed that the descriptors SdsCHE-index, MMFF_29, SssssCcount, DeltaEpsilonC, and T_2_2_1 play an important role in determining the anticancer activity. The positive and negative contribution of these descriptors was showing through the bar graph in Fig. S3B.

2 $$\begin{array}{rcl}{\bf{Predicted}}\,{\bf{L}}{\bf{o}}{\bf{g}}\,{\bf{I}}{{\bf{C}}}_{{\bf{50}}}({\boldsymbol{\mu }}{\bf{M}}) & = & -0.6407\times {\rm{SdssCE}}-{\rm{index}}\\  &  & -0.0336\times {\rm{T}}\_2\_2\_2\\  &  & -0.1278\times {\rm{H}}-{\rm{AcceptorCount}}\\  &  & +0.2226\times {\rm{SdsCHE}}-{\rm{index}}\\  &  & +0.5877\times {\rm{T}}\_{\rm{O}}\_{\rm{O}}\_3\\  &  & +0.5940\end{array}$$

Where,

**SdssCE-index:** Electrotopological state indices for a number of carbon atom connected with one double and two single bonds.

**T_2_2_2:** count of a number of double bounded atoms.

**H-AcceptorCount:** Number of hydrogen bond acceptor atoms.

**SdsCHE-index:** Electro topological state indices for a number of –CH group connected with one double and one single bond.

**T_O_O_3:** Count of a number of Oxygen atoms.

#### QSAR Model 3

The derived QSAR model generated a good activity–descriptors relationship accuracy of 86% referred by regression coefficient (r^2^ = 0.86). The model exhibit internal (q^2^) and external (pred_r^2^) predictive ability of 83% each. The fitness plot for the training and test set was provided in (Fig. 1C). Consecutively a radar plot was provided in Fig. S5 which represents the closeness between the actual and predicted activity of training and test set compounds. The model (equation 3) showed that three descriptors were highly correlated with the biological activity, such as T_T_N_4, T_O_O_3, and SssssCE-index. All the descriptors were directly proportional to the activity and were presented through a bar graph in Fig. S3C.

3 $$\begin{array}{rcl}{\bf{Predicted}}\,{\bf{L}}{\bf{o}}{\bf{g}}\,{\bf{I}}{{\bf{C}}}_{{\bf{50}}}({\boldsymbol{\mu }}{\bf{M}}) & = & +0.0948\times {\rm{T}}\_{\rm{T}}\_{\rm{N}}\_4\\  &  & +0.5217\times {\rm{T}}\_{\rm{O}}\_{\rm{O}}\_3\\  &  & +0.3687\times {\rm{SssssCE}}-{\rm{index}}\\  &  & +1.1313\end{array}$$

Where,

**T_T_N_4**: Topological descriptor for the number of atoms separated from any nitrogen atom by 4 topological bonds.

**T_O_O_3**: Count of number of Oxygen atoms (single, double or triple bonded) separated from the oxygen atom by 3 bond distances in a molecule.

**SssssCE-index**: Electrotopological state indices for number of carbon atom connected with four single bonds.

#### Validation of the developed models

Validation is a very important process for QSAR models. Therefore, various types of validations were procured and the best model was selected by applying different statistical parameters. All the models were successfully validated by using a random test set compounds (Tables S4–S6), along with other parameters such as r^2^, (LOOcv) q^2^, pred_r^2^, Df, F test, best_ran_r^2^, best_ran_q^2^, alpha_test, r^2^ se, q^2^ se, pred_r^2^ _se, ZScore, Applicability domain etc. and the result was provided in Table 1. The r^2^ which necessary to be ≥0.7 was calculated as 0.87 for model 1 and 2, whereas 0.86 for model 3. The result shows that the q^2^ & pred_r^2^ values were ≥0.5 for all the models. The Df were found to be 43, 48 and 35 and the F test was found to be 56.84, 63.17 and 72.59, whereas the Z score was found to be 13.74, 15.84 and 14.51 respectively for Model 1, model 2 and model 3 Table 1. The best_ran_r^2^ & q^2^ are found to be 0.24 and 0.00 for model 1, whereas 0.26 and 0.03 for model 2 and 0.29 and 0.15 for model 3. All the models show 0.00 as the alpha test. The standard error (se) was calculated to identify the possible error in the model’s predicted accuracy. The result shows that the error for r^2^, q^2^ and pred_r^2^se were 0.18, 0.22, and 0.21 respectively, for model 1 and 0.15, 0.17 and 0.21 for model 2 and 0.12, 0.14 and 0.15 for the model 3 (Table 1). The residual error plot of the respective model was shown in Fig. S6. The result shows that the developed models were robust.

**Table 1:** Different statistical validation of the developed QSAR model.

Parameters	QSAR Model 1	QSAR Model 2	QSAR Model 3
r^2^	0.87	0.87	0.86
(LOOcv) q^2^	0.81	0.84	0.83
pred_r^2^	0.82	0.90	0.83
Df	43.0	48.0	35.0
F test	56.84	63.17	72.59
best_ran_r^2^	0.24	0.26	0.29
best_ran_q^2^	0.00	0.03	0.15
alpha_test	0.00	0.00	0.00
r^2^ se	0.18	0.15	0.12
q^2^ se	0.22	0.17	0.14
pred_r^2^ _se	0.21	0.21	0.15
ZScore	13.74	15.84	14.51
Applicability domain	0	0	0

### Virtual designing and filtering of novel GX derivatives

#### Designing of compounds

A library set of about 1000 compounds was designed by using the useful descriptors identified through the QSAR models. Out of 1000 designed xanthone derivatives, 350 compounds were designed based on structural feature selection through A549 cancer cell line specific QSAR model, 350 compounds were designed based on feature selection through HepG2 cell line specific QSAR model, and 300 derivatives were designed based on feature selection through U251 cell line specific QSAR model. Further, these compounds are predicted for its anticancer activity by using the respective models. The compound which has the IC_50_ value of more than 20 µM has been excluded and the rest are carried for additional studies (Tables S10–S12).

#### Screening through Lipinski’s Rule of Five, ADMET Risk and synthetic accessibility

The possible active compounds were further screened through Lipinski’s rule of five for oral bioavailability, which screened out 300 designed compounds. Further, screened compounds were analyzed through pre-ADMET risk screening study. For this, a score based risk was calculated to identify the real ADMET problem behind the compound so that to prevent later failure of the compound. The compounds with an ADMET risk of 10 or more were excluded. The result of candidate compounds 1G along with control compound was shown in Table 2. The result indicates that the 1G showed the risk of 7.3 in comparing to control drug topotecan, which shows a score of 2.0. The risk parameters evaluated were size, charge, water solubility, the volume of distribution, acute rat toxicity and carcinogenicity, SGOT elevation, hepatotoxicity and inhibition of 3A4 oxidation of midazolam (Table 2). The query compounds were screened for their synthetic accessibility. To measure this, the complexity of the ring system, complexity of the molecular structure, similar to commercially available compounds, the number of stereocenters and the potential for using important synthetic reactions were independently weighted to provide a single value for the synthetic accessibility score. Those compounds which show the high score was removed. The Synthetic accessibility score of compound 1G was found to be 7.4 (Table 2).

**Table 2:** The ADMET risk parameters for compound 1G and Topotecan.

Risk	Absorption	P450 oxidation	Mutagenicity	Toxicity	ADMET Risk	Risk Parameters	Synthetic accessibility score
Range	0–8	0–6	0–4	0–7	0–24		
1G	1.48	1.0	1.0	4.0	7.3	Size, Charge, water solubility, Volume of distribution, acute rat toxicity, carcinogenicity in rat SGOT, hepatotoxicity, inhibition of 3A4 oxidation of midazolam	7.41
Topotecan	0.0	0.0	2.0	2.0	2.0	Hepatotoxicity, inhibition of 3A4 oxidation of midazolam	4.23

### Fragment inhibitor screening through molecular docking

**Figure 2:**
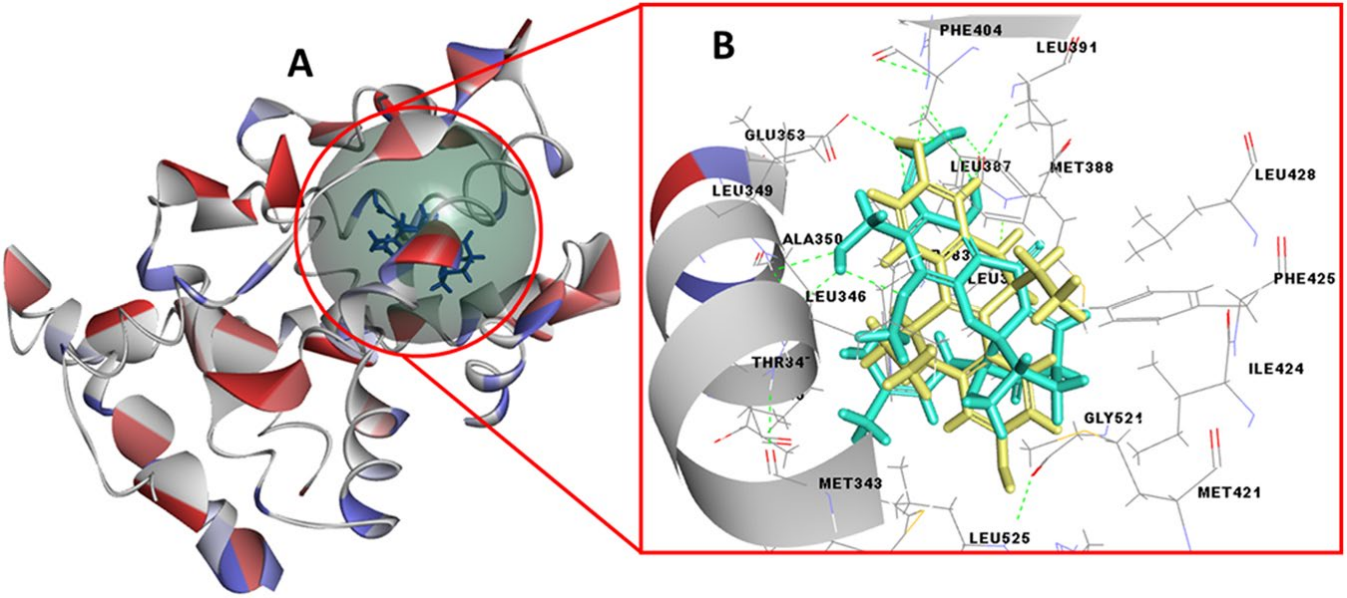
(**A**) Structural model of ESR1 (PDB ID: 1L2I) with ligand binding site (green sphere). (**B**) Binding site pocket residues with best fit confirmation and superimposition of ligand 1G (green) in comparison with control compound ETC (yellow).

The filtered compounds were advanced for docking studies. Prior to docking studies the targets were identified by using the MetaDrug^TM^ program, which resulted in the identification of possible target such as FGF [PDB ID: 4RWJ], ESR [PDB ID: 1L2I], GCR [PDB ID: 4LSJ], CREBP1 [PDB ID: 4TS8], STAT1 [PDB ID: 1BG1], STAT3 [PDB ID: 3CWG] and STAT5 [PDB ID: 1Y1U] (Fig. S1). Later, molecular docking study was performed on these identified target receptors for filtered compounds and by using control (co-crystallized inhibitors in complex) namely, C1 (66 T/CID:51039095), C2 (ETC/CID:4474781), C3 (LSJ/CID:72710581), C4 (XZ8/CID:6937521), C5 (PTR/CID:30819), C6 (CID:160254), and C7 (CID:2726045), respectively (Fig. S7). After scrutinizing all the results, the top hit compound 1G were explored and presented in detail. The best docking score for compound 1G was achieved against the ESR1 receptor. The docking results for most promising compound 1G on target ESR1, showed a high binding affinity as indicated by LibDock (docking) score of 121.33 and binding energy of −154.27 kcal/mol, in comparison to co-crystallized inhibitor ETC, *i.e*., LibDock score of 112.22 and binding energy of −103.55 kcal/mol (Table 3). Docking results revealed the presence of both H-bonds and hydrophobic interactions within the docked binding site of ESR1 (Fig. 2A). The compound 1G when docked showed several pose and orientation and thus several configurations. Each configuration is combined score of Vander Waals forces, H-bonds, pi interactions and other parameters and refers in form of LibDock score. Higher the LibDock score means a high chance of ligand-protein binding. Results of docking poses and binding mode conformations revealed that HH_21_ and HH_22_ of Arg_394_ (basic, polar, positive charged), donate the hydrogen atom which was accepted by O_29_ atom of compound 1G. Whereas, compound 1G donates atom (H_39_) and (H_52_) which were accepted by an oxygen atom of Leu_346_ (hydrophobic), and Phe_404_ (hydrophobic, aromatic).

**Table 3:** Details of LibDock scoring functions, H-bond, binding energy and interacted binding site amino acid residues for compound 1G & control drug docked on anticancer target ESR1.

S.No.	LibDock Score	H bonding	Binding energy (kcal/mol)	PLP1	Jain score	Ludi	-PMF	Interactive Amino acid residue
1G	121.33	ARG_394_ (2) LEU_346_ PHE_404_	−154.27	99.46	6.83	629	157.7	MET_343_, LEU_346_, THR_347,_ LEU_349_, ALA_350_, GLU_353_, TRP_383_, LEU_384_, LEU_387_, MET_388_, LEU_391_, ARG_394_, PHE_404_, MET_421_, ILE_424,_ PHE_425_, LEU_428_, LEU_525_, LEU_540_
ETC (Control)	112.22	ARG_394_ GLU_353_	−103.55	92.85	6.02	674	135.9	MET_343_, LEU_346_, THR_347_, LEU_349_, ALA_350_, GLU_353_, TRP_383_, LEU_384_, LEU_387_, MET_388_, LEU_391_, ARG_394_, LEU_402_, PHE_404_, MET_421_, ILE_424_, PHE_425_, LEU_428_, GLY_521_, HIS_524_, LEU_525_, LEU_540_

These hydrogen bonds may be considered as a stabilizer of the complex and may be the reason for conformational stability and thus resulted in significant activity (Fig. 2B). The interactive amino acid residues were presented in Table 3. These interactions were displayed with 2D diagram and represented by different colors *e.g*., pink indicates electrostatic interactions; purple indicates the covalent bond, and green indicates Van der–Waals interaction. Solvent accessibility of the ligand atoms and the amino acid residues are shown in a light blue shade surrounding the atom or residue. High shade indicates more exposure to the solvent (Fig. S8). The measured binding affinity of compound 1G and the inhibitor of ESR1 receptor in terms of the LibDock score was further re-calculated through different other scoring functions so that to avoid false positive predictions. The calculated docking scores of compound 1G against ESR1 were 99.46, 6.83, 629 and 157.7 for PLP1, Jain, Ludi and PMF scoring functions, respectively. However, for control drug/inhibitor of ESR1, the calculated docking scores were 92.85, 6.02, 674 and 135.86 for PLP1, Jain, Ludi and PMF scoring functions, respectively. These results indicate that compound 1G docking scores were higher than the control drug/inhibitor (Table 3). These results may provide a molecular level of the foundation, to understand the possible mode of action of top hit compound 1G. This infers that compound 1G may be a potential inhibitor of ESR1.

### Compliance with *in silico* pharmacokinetics parameters

The use of in silico methods to predict various pharmacokinetics parameters such as ADMET is intended as a first step to analyze the novel chemical entity, to prevent wasting time on lead candidates that would be toxic or metabolized by the body into an inactive form and unable to cross membranes. In the studied work, different physicochemical properties of the candidate compound 1G were calculated and then compared with that of standard drug topotecan. The calculated molecular diffusion coefficient in water for compound 1G was 0.58, and octanol-water distribution coefficient (LogP/D) was 3.45. The compound 1G was found slightly lipophilic in nature and showed good solubility, but slightly lower than standard anticancer drug topotecan. Results of compound 1G showed a tendency to supersaturate in water, with a native water solubility of 7.87E^−03^ mg/mL. The solubility of the compound 1G in the fasted state at gastric fluid was found 2.11E^−03^ mg/mL, and in a fasted state intestinal fluid, it was 1.86E^−02^ mg/mL, whereas, in a fed state intestinal fluid, it was found 1.16E^−01^ mg/mL. The compound 1G was analyzed for permeability measurement through human skin and human jejunal effective permeability, along with apparent MDCK COS (Madin-Darby Canine Kidney, Cells-On-Sheet) permeability, and permeability through rabbit cornea. The calculated permeability through human skin was 24.09 cm/s × 10^7^ and for Peff, it was 2.47 cm/s × 10^4^ cm/s × 10^7^. The calculated MDCK permeability for compound 1G was 381.31 cm/s × 10^7^, and the permeability through the rabbit cornea was 67.35 cm/s × 10^7^. These results indicate high intrinsic passive uptake capacity of the liver, which considered good in the sense of pharmacology studies.

The calculated volume of distribution of compound 1G was 5.33 L/kg. The compound 1G showed the ability to cross the BBB partition. The brain/blood partition coefficient was found (in logarithm) −0.36, whereas the percent unbound to blood plasma proteins was 4.97. The predicted blood to plasma concentration ratio was 0.72 for Compound 1G. The major CYPs involved were CYP3A4, CYP1A2, CYP2C9, and CYP2C19, and the compound 1G showed potency to inhibit these CYPs. The compound 1G was predicted to act as a substrate of CYP3A4, and the identified sites of metabolism on compound 1G were C13, C16, C21, and C24. The compound 1G was found to be the inhibitor of the CYP3A4-mediated metabolism of midazolam and testosterone. The calculated inhibition constant (K_i_) valuations for midazolam and testosterone inhibition were 0.769 and 69.033 µM, respectively. The affinity of compound 1G for CYP450 enzymes in quantitative terms was also evaluated. The calculated K_m_ and V_max_ values provide the knowledge of metabolic rate. The calculated kinetics Michaelis-Menten K_m_ constant for predicted sites of CYP3A4 mediated metabolism was 1.89E + 01 µM, whereas the calculated V_max_ constant for predicted sites of enzyme CYP3A4 mediated metabolism was 1.40E + 01 nM/min/nM. The calculated intrinsic clearance constant (CL_int_) for predicted sites of CYP3A4 mediated metabolism was 8.25E +01 µL/min/mg. The enzyme kinetics Michaelis-Menten K_m_ constant value for CYP3A4 mediated metabolism (human liver microsomes) was 2.36E + 02 µM, while V_max_ constant for predicted sites of CYP3A4 mediated metabolism (human liver microsomes) was 6.70E-01 nM/min/nM. The intrinsic clearance constant for predicting sites of CYP3A4 mediated metabolism (human liver microsomes) was 2.84E + 00 µL/min/mg. These metabolisms related enzyme kinetics data may further be used to calculate the hepatic clearance and *in vitro*/*in vivo* relationship. The overall calculated intrinsic clearance in human liver microsomes was 1.82E^+01^ and 1.64E^+01^ μL/min/mg for compound 1G and standard anticancer drug topotecan, respectively. These results suggest that the renal clearance may decrease for compound 1G, but metabolic clearance may increase. Through this metabolic rate, a precise knowledge of elimination rate may be optimized and later can be used to calculate the drug’s half-life and total clearance (Tables S13 and S14).

**Figure 3:**
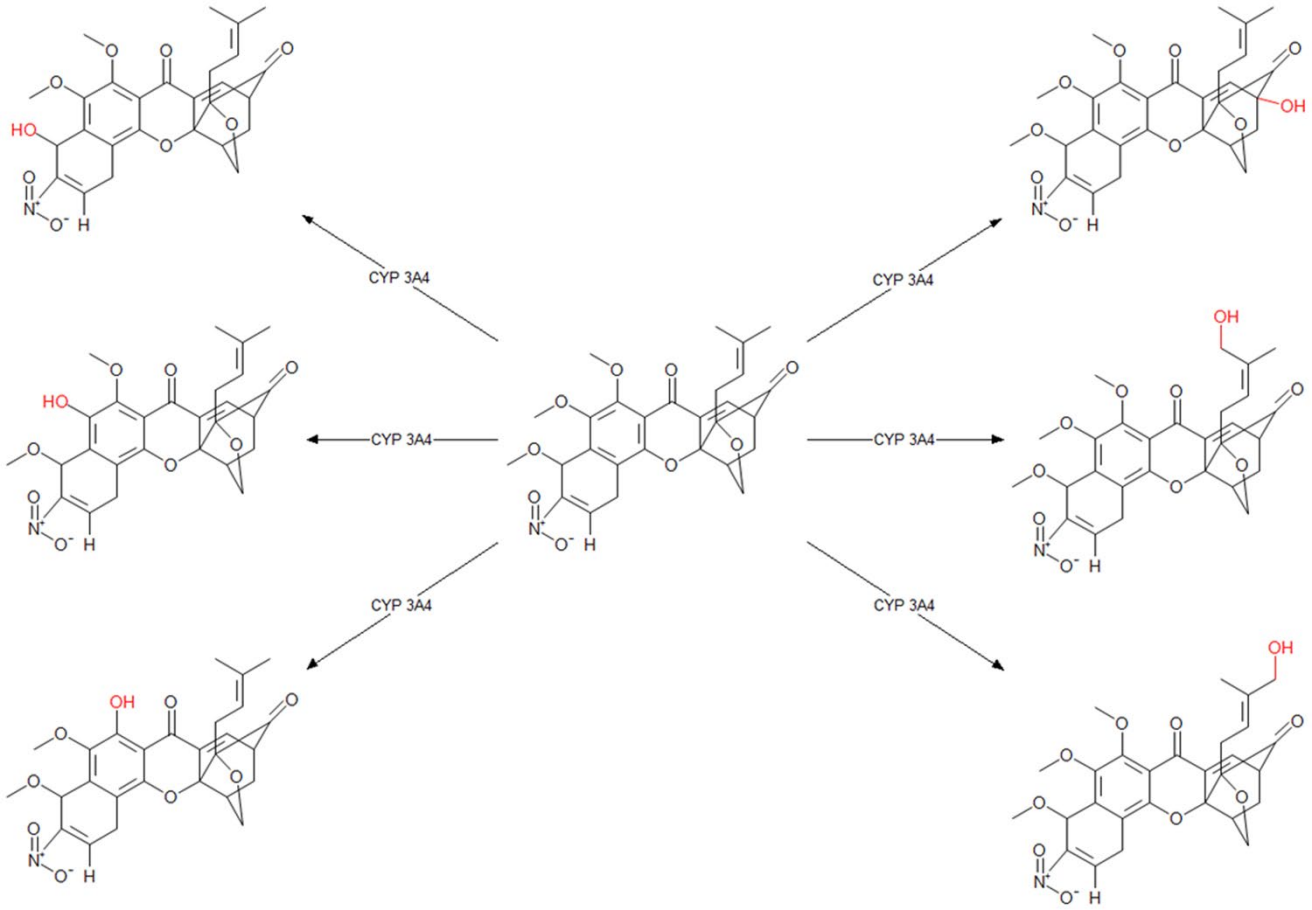
The predictive metabolites and sites of metabolism of candidate compound 1G.

**Figure 4:**
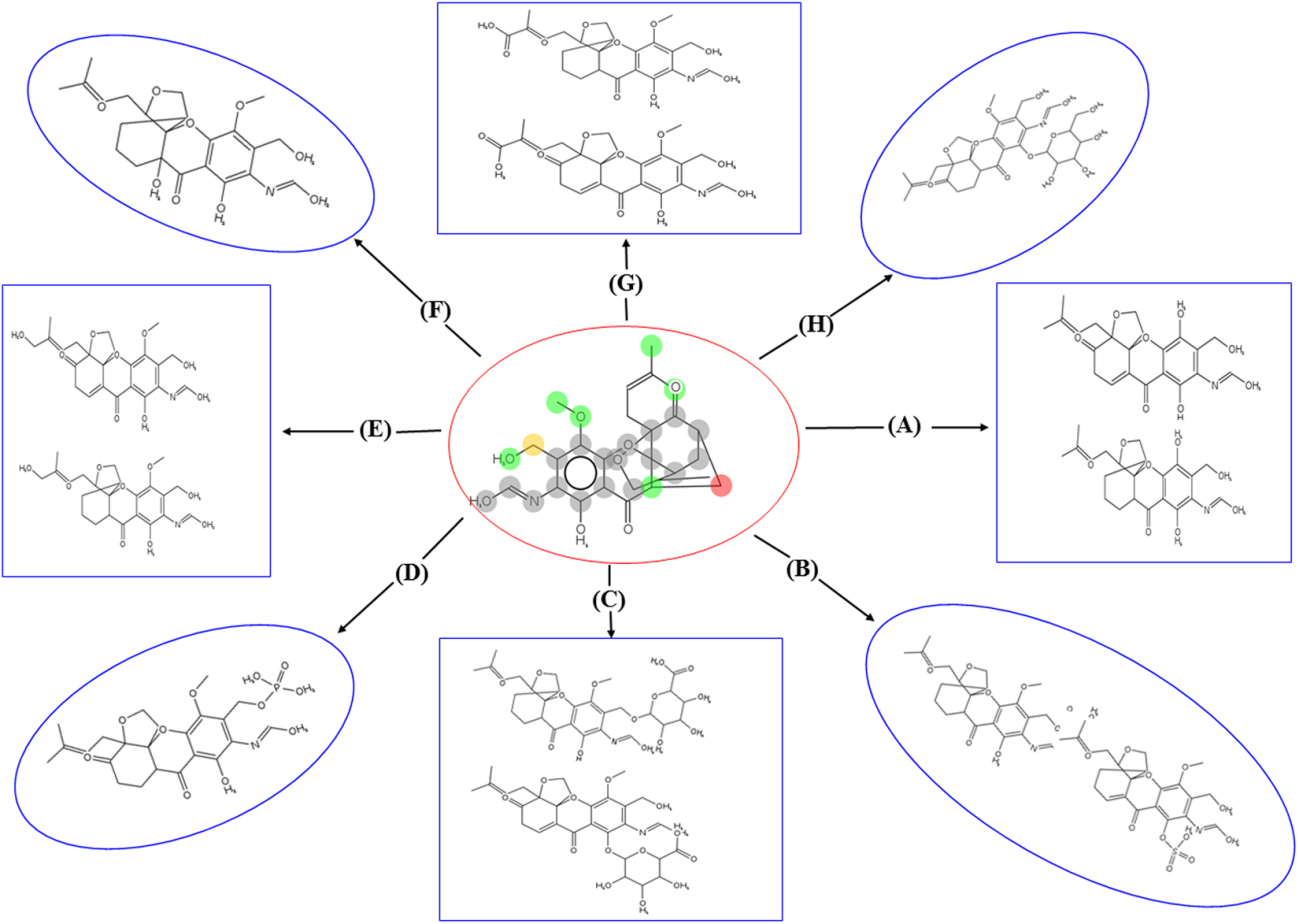
The possible reactions and metabolites of Compound 1G. (**A**) Demythalation (**B**) Sulfation, (**C**) Glucuronidation, (**D**) Phosphorylation, (**E**) Hydroxylation, (**F**) Hydration, (**G**) Oxidation, (**H**) Glucosidation.

In addition to the CYP450, however, there are a variety of other drug metabolizing enzymes such as oxidases, hydrolases, reductases, and dehydrogenases (oxidoreductases) that can affect the distribution of orally administered compound in the systemic circulation. To study potential metabolism mediated compound interactions in terms of their metabolites, have important implications for both drug efficacy and safety. For this, compound 1G was analyzed computationally for investigation of possible drug metabolism and their metabolites implications. Due to this, predicted results of potential metabolic sites, metabolites, and type of reactions involved for compound 1G are discussed here in details. Results of CYP450 mediated metabolism of compound 1G showed the probability of six possible metabolites by CYP 3A4 enzyme (Fig. 3). Moreover, results also showed the possible metabolic sites and corresponding metabolites for each biochemical reaction. These metabolic reactions for compound 1G were demethylation, glucuronidation, oxidation, sulfation, glucosidation, phosphorylation, and hydration (Fig. 4). However, no sites were identified for (UGT) uridine 5′-diphospho-glucuronosyl transferases family, which catalyze in Phase II metabolism and has the potential to transform small molecules to water-soluble form. The overall results suggest that compound 1G covers, good drug-like properties.

#### Molecular interactions of compound 1G with druggable proteins

There are some proteins which are reported to be involved in xenobiotic metabolism, detoxification, BBB penetration and channel inhibition. Results indicate that compound 1G may activate the PXR, which regulates the expression of proteins involved in detoxification. Beside this, compound 1G showed the potential to become a substrate, as well as possesses a tendency to inhibit the human P-glycoprotein transporter (Pgp), which is known to be involved in multiple drug resistance (MDR) and BBB penetration. Results also indicate that compound 1G inhibited by the hepatic organic anion-transporting polypeptide (OATP-1B) transporter, thus there may be a little chance of drug–drug interaction (Table S13). These results support the predicted anticancer activity of compound 1G.

#### Predicted toxicology of compound 1G

Results of *in silico* toxicity risk assessment for compound 1G revealed no sign of cardiotoxicity and anemia, similar to standard anticancer drug topotecan. Results of compound 1G showed no hERG (human ether-a-go-go-related gene) potassium channel inhibition in human, similar to standard drug topotecan, which otherwise may cause risk of cardiotoxicity. However, results showed that compound 1G may cause mild nephrotoxicity if used for prolonged or at high doses. Drug-induced liver injury (DILI) studies indicate that compound 1G may elevate the level of GGT, SGOT, SGPT, ALP and thus may cause liver necrosis, Cholestasis and may damage the bile duct, On the other hand, level of LDH enzymes thus not cause any risk of ischemic hepatitis. Likewise, results of compound 1G showed predicted rat maximum tolerated dose in feed state was 0.16 g/kg body weight, similar to standard drug topotecan i.e., 0.15 g/kg body weight. The compound 1G predicted to be non-sensitized against toxicity risk parameter allergenic respiratory sensitization in the rat, similar to topotecan. Beside this, compound 1G showed moderate ocular irritancy, mild skin irritant, and weak skin sensitization, in contrary to topotecan, which showed none irritancy, except severe ocular irritancy. However, compound 1G was detected non-toxic for estrogen receptor toxicity in the rat, while topotecan showed a toxic response. On the contrary, compound 1G showed toxic response against androgen receptor toxicity in the rat, similar to topotecan. However, both 1G and topotecan cause non-toxic response against parameter causing phospholipidosis. The compound 1G showed non-mutagenic (Ames) response, similar to topotecan. In terms of pure compound and metabolites, the predicted mutagenicity responses varied against different strains of *S. typhimurium* (Table 4). The compound 1G may cause developmental toxicity, similar to topotecan. No carcinogenicity potential predicted for both female and male species of rat and mice. The carcinogenic potency for compound 1G in terms of predicted tumorigenic dose, 50% (TD_50_) for the mouse and rat were 4.33 and 2.60 mg/kg body weight/day, respectively, similar to topotecan (Table 4). These toxicity risk assessment results overall indicate that compound 1G showed under limit toxicity range, except few parameters, which can be optimized by dose limit experiments. These results will be helpful in setting dose ranges for *in vivo* small animal’s assays.

**Table 4:** Details for calculated toxicity risk parameters for compound 1G and control drug topotecan.

Compound		1G	Topotecan
Cardiotoxicity		Non Toxic	Non Toxic
Anaemia		No	No
Nephrotoxicity		Mild Toxic	Non Toxic
Hepato-toxicity	levels of Alkaline Phosphatase enzyme	Elevated	Elevated
	levels of GGT enzyme	Elevated	Normal
	levels of LDH enzyme	Normal	Normal
	levels of SGOT enzyme	Elevated	Elevated
	levels of SGPT enzyme	Elevated	Normal
pIGC_50_ for Tetrahymena pyriformis growth inhibition toxicity		1.688	0.754
hERG potassium channel inhibition in human		No	No
Estrogen receptor toxicity in rats		Nontoxic	Toxic
Androgen receptor toxicity in rats		Toxic	Toxic
Allergenic respiratory sensitization in rat		Non-sensitizer	Non-sensitizer
Causing phospholipidosis		Nontoxic	Nontoxic
Rat Inhalational LC_50_		0.185949 mg/m3/h	9.10882 mg/m3/h
Rat Maximum Tolerated Dose feed (g/kg_body_weight)		0.161302	0.150435
Developmental Toxicity Potential		Toxic	Toxic
Carcinogenic Potency TD_50_ (mg/kg_body_weight/day)	Mouse	4.32844	1.02803
	Rat	2.60491	7.50803
FDA Rodent Carcinogenicity	Mouse female	Non-Carcinogen	Non-Carcinogen
	Mouse male	Non-Carcinogen	Non-Carcinogen
	Rat female	Single-Carcinogen	Single-Carcinogen
	Rat Male	Non-Carcinogen	Single-Carcinogen
Daphnia EC_50_		2.96487 mg/l	30.8959 mg/l
Rat Chronic LOAEL (g/kg_body_weight)		0.00875437	0.0021976
Aerobic Biodegradability		Degradable	Non-Degradable
Ocular Irritancy		Moderate	Severe
Skin Irritancy		Mild	None
Skin Sensitization		Weak	None
Triggering the mutagenic chromosomal aberrations		Toxic	Toxic
mutagenicity (pure compound)	Ames	Non-Mutagen	Non-Mutagen
	TA97 and/or TA1537 strains of S. typhimurium	Negative	Positive
	TA98 strain of S. typhimurium	Positive	Negative
	TA100 strain of S. typhimurium	Negative	Negative
	S. typhimurium and/or WP2 uvrA strain of *E. coli*	Negative	Positive
	TA1535 strain of S. typhimurium	Negative	Negative
mutagenicity (microsomal rat liver metabolites)	TA97 and/or TA1537 strains of S. typhimurium	Positive	Positive
	TA98 strain of S. typhimurium	Positive	Negative
	TA100 strain of S. typhimurium	Negative	Negative
	TA102 strain of S. typhimurium and	Negative	Negative
	TA1535 strain of S. typhimurium	Negative	Negative

Abbreviations: EC_50_, effective concentration 50%; FDA, Food and Drug administration; LC_50_, lethal concentration 50%; LD_50_, lethal dose 50%; LOAEL, lowest observed adverse effect level; TD_50_, tumorigenic dose 50%; SGOT, serum glutamic oxaloacetic transaminase; SGPT, serum glutamate-pyruvate transaminase; GGT, gamma glutamyl transpeptidase; LDH, lactate dehydrogenase.

### Enrichment analysis through Systems Pharmacology

To find out the processes upregulated or downregulated by compound 1G, function-based pathways, compounds association analysis, GO terms were studied in detail, through biological processes, molecular functions, and genes set enrichment scores. This study resulted in a relationship between the effect of compound 1G and biological processes. These EA results for compound 1G are represented through the GO process and key network object (Fig. S9), metabolic networks (Fig. S10), and biological process networks (Fig. S11) and corresponding biological pathway maps. The significance of EA score was represented by p-value (negative logarithm; -log value). Higher p-value represents higher relevance of the entity. Thus, each process ranked based on quantitative p-value and so summarizes the pharmacological and toxic effect at systems pharmacology level.

#### GO Processes & Key Network Objects

The key network objects and GO processes were identified for compound 1G through the MetaDrugTM database tool. A total of 27 networks was predicted, however, the top networks were selected based on g-score. Highly positive g-score means, the network is highly saturated with genes (Table 5). The key networks identified are glutathione s-transferase (GSTA1), sulfotransferase (SULT1A1), huntingtin interacting protein (HYEP), N-acetyltransferase (NAT-1), aryl hydrocarbon receptor nuclear translocator (ARNT2) and the major GO Processes identified was responding to xenobiotic stimulus & xenobiotic metabolic process. A canonical pathway for the top scored network (based on a number of pathways) from active experiments are shown in Fig. S12.

**Table 5:** The key network objects and GO processes along with total nodes.

No	Key network objects	GO Processes	Total nodes	p-Value	g-Score
1	GSTA1, SULT1A1, HYEP, NAT-1, ARNT2	Response to xenobiotic stimulus (69.2%), xenobiotic metabolic process (61.5%), cellular response to xenobiotic stimulus (61.5%)	51	3.01e-38	61.77

#### Identified metabolic networks for compound 1G

The drug-induced metabolic changes in the metabolic networks are identified and score in the term of −log (p-value) score. Through this score, a subset of metabolic networks was identified and ranked in Fig. S10, which seems more influenced by the compound 1G. These changes were due to the interactions of some regulatory proteins with the compound 1G or its metabolites. The result indicates that the compound 1G more interferes with the Estradiol & Estrone metabolism. The details interfere are shown here with the pathway details.

#### Analysis of Estradiol metabolism influence by compound 1G

**Figure 5:**
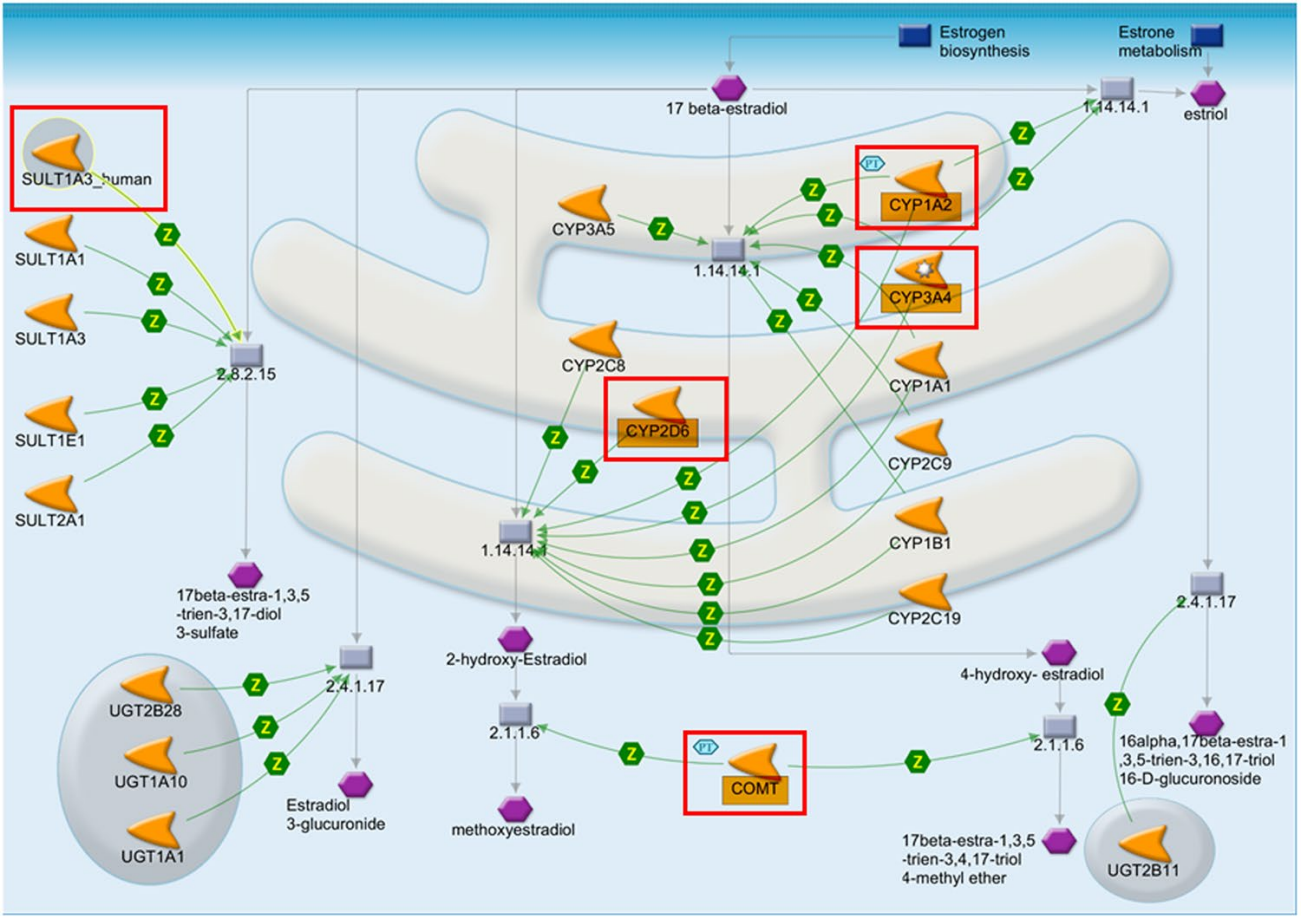
Representing the pathway maps for Estradiol metabolism influence by compound 1G. The red square represents the affected proteins.

In this pathway, the major cytochrome involved were CYP1A2, CYP3A4, and CYP2D6. They catalyze the intermediate enzyme monooxygenase, which hydroxylated the 17β-estradiol into three different endogenous metabolites *i.e*., 2-hydroxyestradiol, 4-hydroxyestradiol, and estriol. Consequently, COMT (catechol-O-methyltransferase) catalyzes and convert 2-hydroxy-estradiol into methoxy estradiol and 4-hydroxyestradiol into 17-β-estra-1, 3, 5-trien-3, and 17-triol-4-methyl ether. The important transporter SULT1A3 catalyzes and converts the 17β estradiol into endogenous metabolites 17β-estra-1, 3, 5-trien-3, and 17-diol-3-sulphate. The UGT2B28, UGT1A10, and UGT1A1 catalyzes the which results in estradiol 3-glucuronide. Whereas the UGT2B11 catalyzes and convert the estriol into 16-α, 17-β-estra-1, 3, 5-trien-3, 16, 17-triol-16-D-glucuronoside (Fig. 5).

**Figure 6:**
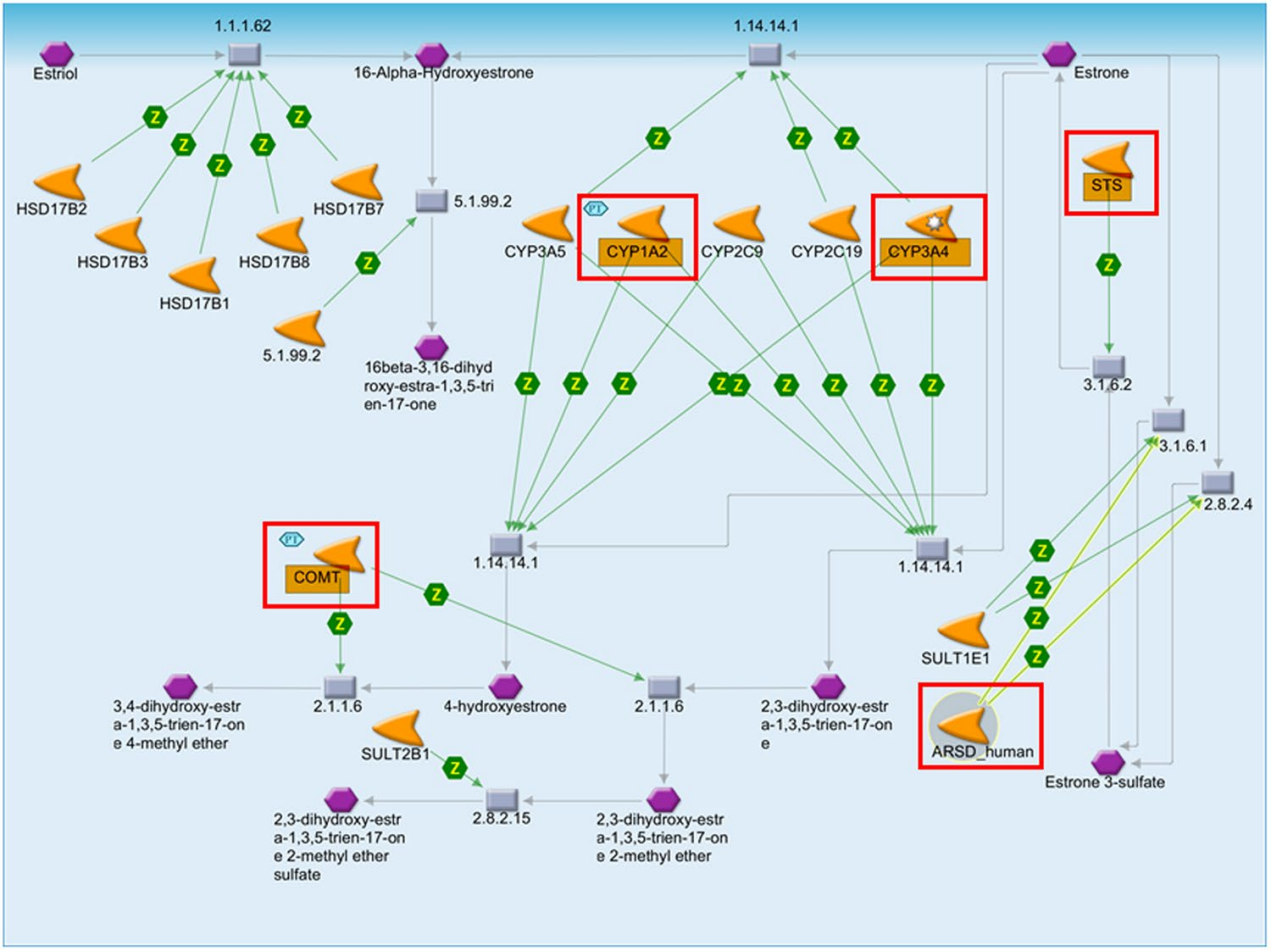
Representing the pathway maps for estrone metabolism influence by compound 1G. The red square represents the affected proteins.

Similarly, in estrone pathway its identified that CYP1A2 and CYP3A4 reacts with estrone and convert it into three endogenous metabolites, i.e. 16-α-hydroxyestrone, 4-hydroxyestrone and 2,3-dihidroxy-estra-1,3,5-trien-17-one. On the other hand the Arylsulfatase (ARSD) catalyze the reaction and convert estrone into estrone-3-sulfate, which was later re-converted into estrone by catalyzing by **s**teryl-sulfatase (STS) enzyme. Simultaneously the COMT catalyzes, and convert 4-hydroxyestrone & 2,3-dihidroxy-estra-1,3,5-trien-17-one and convert them into 3, 4-dihidroxy-estra-1,3,5-trien-17-one-4-methyl-ether and 3-dihidroxy-estra-1,3,5-trien-17-one into 2,3-dihidroxy-estra-1,3,5-trien-17-one-2-methyl-ether respectively (Fig. 6).

#### Biological process networks analysis for compound 1G

Through the biological process network analysis, it’s identified that three biological processes i.e. bile acids transport system, bile acid regulation of lipid metabolism and negative FXR-dependent regulation of bile acids concentration and regulation of drug metabolism through neurophysiological process circadian rhythm was mainly affected by 1G (Fig. S11). The studies of these networks will help to find a meaningful relationship between biological processes and lead compound, prior to experimental evaluation of small animals or clinical trials.

#### Bile acid transport system analysis and its regulation

The result shows that the extracellular and intracellular bile acid binds with the PXR, which transcriptionally regulate the OST-β (organic solute transporter beta) major basolateral bile acid transport system and MDR1, MDR3 and OATP8. The PXR also binds with BSEP and activate it. The PXR makes a complex subunit of PXR/RXRα. This complex subunit transcriptionally regulates the small heterodimer partner (SHP), and again make a complex subunit RAR-α/RXR-α. This subunit transcriptionally regulates the solute carrier family 10 member 1 (SLC10A1) and hepatocyte nuclear factor (HNF4-α). The SHP also regulates the mono-conjugated bile acid, which later binds with scavenger receptor class B member (SR-BI). The PXR/RXRα also induces the bile acid conjugation enzymes, SULT2A1. This activation of FXR reduces hepatic fat accumulation and the level of plasma triglyceride (Fig. S13).

#### Bile acid regulation of lipid metabolism

The analysis result, direct that the intracellular bile acid bound with FXR and activate it. On the other hand, bile acids go for reaction with CYP7A1 and make a product of bile acid CoA and later into mono-conjugated bile acid. This product has further transformed into di-anionic bile acids. The targeted FXR, transcriptionally regulate UGT2B4, peroxisome proliferator-activated receptor alpha (PPARα), and SULT2A1. This SULT2A1 transcriptionally regulate the mono-conjugated bile acid. Simultaneously the FXR transcriptionally regulates the PXR, a nuclear receptor which makes a complex subunit PXR/RXRα. The complex PXR/RXRα transcriptionally regulates the cytoplasmic enzyme CYP3A4 and CYP2B6. The CYP3A4 activates the SULT2A1 which transcriptionally regulate the mono-conjugated bile acid and catalyzes a reaction which transforms the mono-conjugated bile acid into di-anionic bile acids. The transcriptionally activated PPARα considered to be the important regulator of intra and extracellular lipid metabolism. The activated FXR increase the lipid oxidation. The FXR targeted the PPARα. The FXR/RXRα transcriptionally activate the gastrotropin, this protein can bind bile acid and play roles in fatty acid uptake, transport, and metabolism (Fig. S14). These results indicate that there was modulation of bile acid receptors by compound 1G, which seems functionally active with a role in lipid metabolism, therefore there is little or no risk of drug-induced toxicity on lipid metabolism.

#### Neurophysiological process analysis (Circadian rhythm)

Screening results showed that compound 1G may also affect the circadian rhythm. To demonstrate the affected neurophysiological processes by compound 1G and its metabolites, circadian rhythm analysis was studied and highlighted the molecular insight mechanism of action. The result of this regulation was analyzed and presented through a canonical pathway. The result showed that the initial regulation was by retinoic acid – related orphan receptor (RORα) and reverse-erythroblastosis (Rev-ERBα) which modulate the neuronal pas domain protein (NPAS2). This NPAS2 also regulated by NAD (+). Further, this NPAS2 bind with cryptochrome (CRY2), which transcriptionally regulates brain and muscle ARNT-like protein (BMAL1). Later, BMAL1 binds with the circadian locomotor output cycles kaput (CLOCK) transcription factor. The CRY2, CRY1, BMAL1, and CLOCK all together regulate the period circadian protein (PER3 & PER2) (Fig. S15).

**Figure 7:**
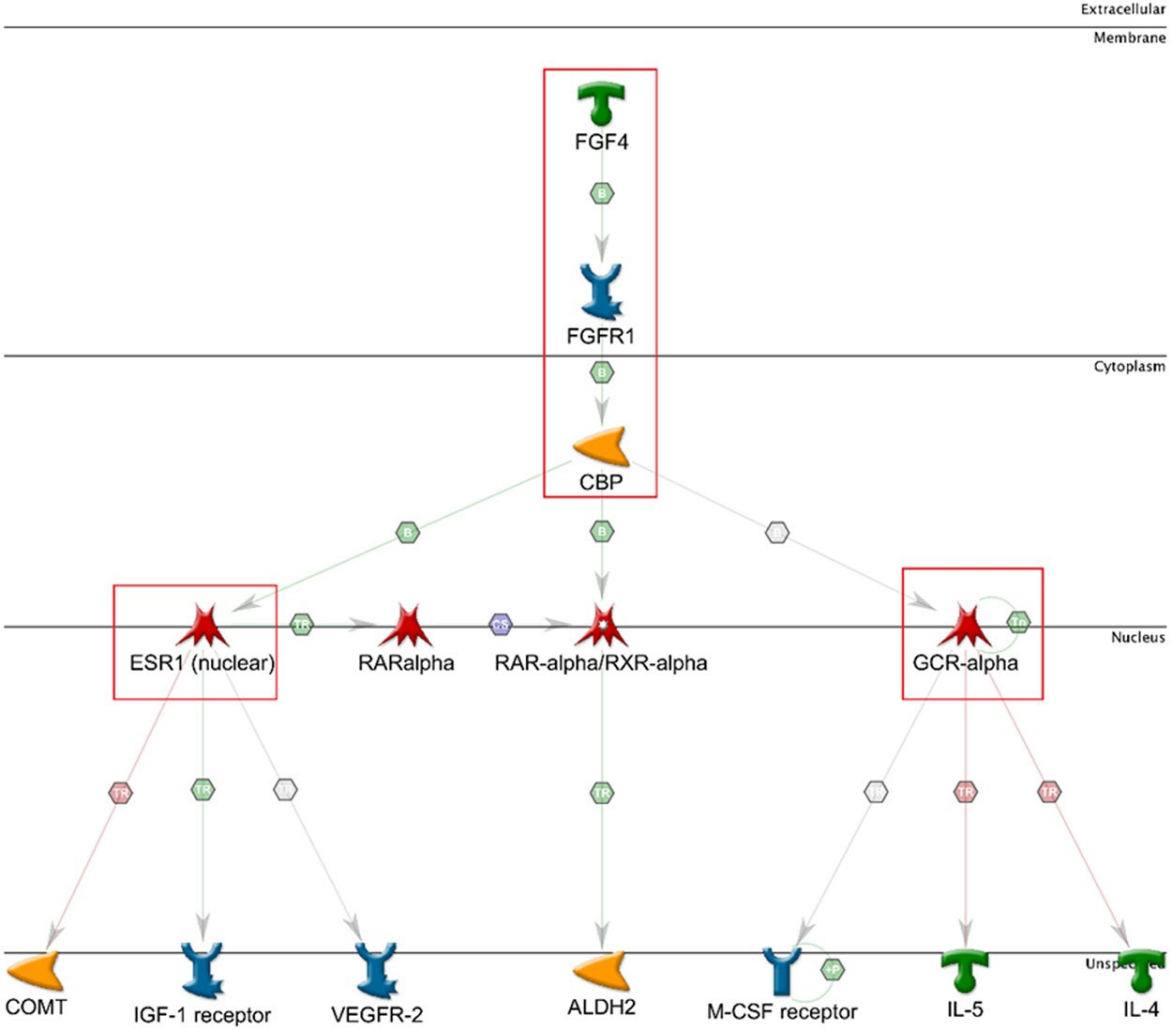
Signal transduction Pathway analysis of the possible drug target FGR, ESR1 and GCRα and their localization. The arrow marks represent the direction and the red encircle shows important targets in pathway.

#### Identification of Drug target networks for compound 1G

The possible mechanism of action of compound 1G was derived by mining the available omics data and thus hypothetically identified the pathway and associated functional genes involved. Results of *in silico* signal transduction studies suggest that compound 1G or its metabolites may interfere cancer cell signaling process through interaction with transporter FGF4 or its cell membrane receptor FGFR1. This interference resulted in induction of transcriptional co-activation enzyme CBP (CREB-binding protein) in the cytoplasm. The CBP enzyme may transduce this signal through three nuclear membrane receptors namely, ESR1, RAR-alpha/RXR-alpha, and GCR-alpha. The ESR1 predicted to regulate nuclear proteins, namely, COMT, IGF-1, and vascular endothelial growth factor receptors (VEGFR-2). For therapeutic activity, ESR1 receptor transcriptionally activates the Insulin-like growth factor 1 (IGF-1), while inhibiting the COMT. In another signaling pathway, CBP may bind to RXR-alpha and transcriptionally activate the aldehyde dehydrogenase 2 family (ALDH2), thus resulted in inhibition of cell proliferation. The other possible target suggested to be GCR-alpha, which transcriptionally inhibit the IL-4, IL-5, and regulate the macrophage colony-stimulating factor (M-CSF) a nuclear receptor, thus resulted in inhibition of cell division (Fig. 7).

#### Off-target prediction for compound 1G

**Figure 8:**
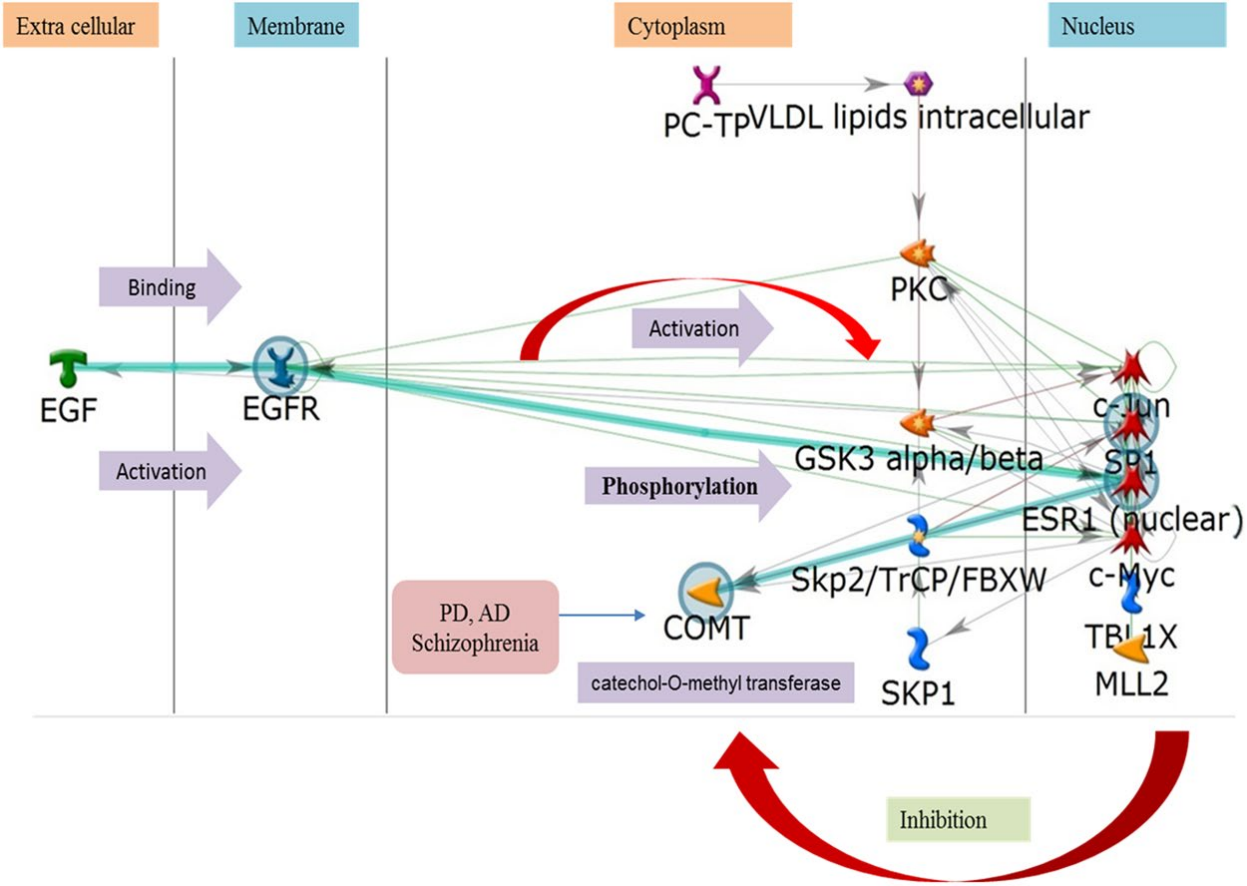
Represneting the ‘on’ and ‘off’ target of the compound 1G with the intermediate signaling pathway. The mechanism of the compound is also describe.

Results suggest that compound 1G may interact and interfere with epidermal growth factor (EGF) which well along bind and activates the cell membrane EGFR receptor, and later activate the nuclear C-Jun and SP1 transcription factors. In response to the activation of these nuclear transcription factors, results suggest that it may cause inhibition of cytoplasmic off-target enzyme COMT (which is a known drug target for Parkinson’s disease (PD), Alzheimer disease (AD), and Schizophrenia (Fig. 8). These off-target results suggest the future scope of compound 1G for the evaluation of anti-PD, anti-AD, and antipsychotic (against Schizophrenia) activity, which is a subject of further research work.

## Conclusion

The quest for high target potency should not be pursued blindly, without an understanding of its relevance to efficacy and efficiency. The strategy used in this study may provide understanding in designing novel and promiscuous Garcinia caged xanthones as anticancer agents. Differentiating and describing the role of important chemical descriptors identified through QSAR modeling gives an idea of key descriptors responsible for the *in vitro* anticancer/cytotoxic activity. *In silico* approaches were used to virtually screen top hit compound 1G, and later validated by evaluation through oral bioavailability parameters, ADMET risk screening, docking, *in silico* pharmacokinetics/pharmacodynamics (PK/PD) screening study, and lastly with systems pharmacology approach. Structure-guided insights of molecular interactions were explored to highlight the multi-level cell networks for biological processes, networks, and signal transduction pathways for Compound 1G. Also highlighted the on and off-targets of Compound 1G. Based on signaling pathways and molecular docking energy, potential targets of compound 1G were suggested. These studied methods can be used as a template work, ahead of smart drug discovery path. By using these QSAR models and through standardization of compounds and centering and scaling of descriptors a set of virtually designed compounds can be predicted as the promiscuous cancer inhibitors.

## Electronic supplementary material

Supplementary Material
