# Supplementary material for: Virtual screening, Docking, ADMET and System Pharmacology studies on Garcinia caged Xanthone derivatives for Anticancer activity
Source: Sci Rep. 2018 Apr 3;8:5524. doi: 10.1038/s41598-018-23768-7 (PMC5883056; doi:10.1038/s41598-018-23768-7)
Supplement: Supplementary file 1 — Supplementary Material [file 41598_2018_23768_MOESM1_ESM.pdf]

Virtual screening, Docking, ADMET and System Pharmacology studies on Garcinia caged Xanthone derivatives for Anticancer activity

Sarfaraz Alam[1, 2] & Feroz Khan[1, 2,3*]

[1]Metabolic & Structural Biology Department, CSIR-Central Institute of Medicinal & Aromatic Plants, P.O.-CIMAP, Lucknow-226015 (Uttar Pradesh), India

[2]Academy of Scientific & Innovative Research (AcSIR), CSIR-CIMAP Campus, Lucknow-226015 (Uttar Pradesh), India

*Correspondence to be addressed:
Metabolic & Structural Biology Department, CSIR-Central Institute of Medicinal & Aromatic Plants, P.O.-CIMAP, Kukrail Picnic Spot Road, Lucknow-226015 (Uttar Pradesh), India; Tel.: +91 522 271 7668, Fax: +91 522 234 2666; E-mail: f.khan@cimap.res.in; CIMAP Communication No.: CIMAP/PUB/2016/84.

[3]Present Address: Skaggs School of Pharmacy & Pharmaceutical Sciences, University of California San Diego (UCSD), 9500 Gilman Drive, La Jolla, San Diego, CA 92093, USA.

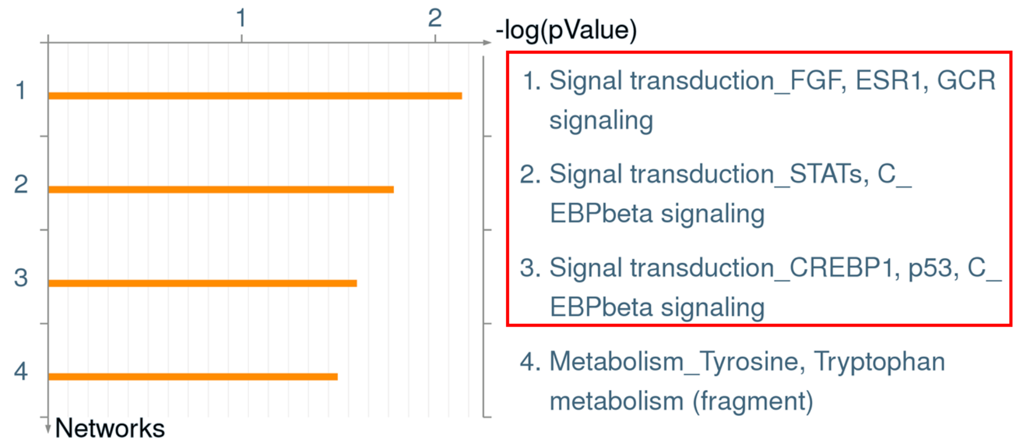

Figure S1: Representing possible target (red square) for the candidate compound and their P value. (Bar length reflects the significance and equals to the negative logarithm of enrichment p-value).

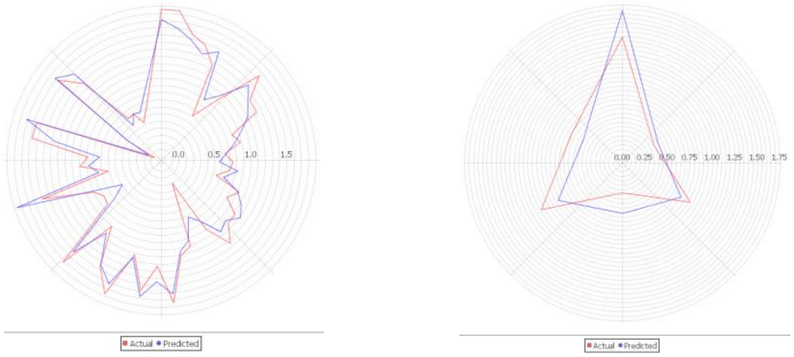

Training Test

Figure S2: A radar plot representing closeness between the actual and predicted activity of the compounds of training and test set of QSAR model 1.

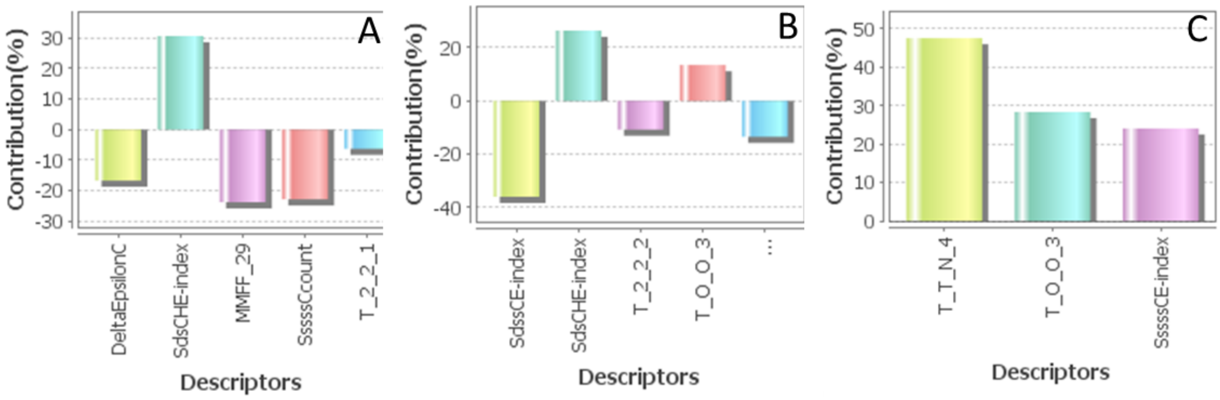

Figure S3: The bar graph showing the contribution of different descriptors that are important for activity variation in the model. (A) QSAR Model**1**; (B) QSAR Model**2**, (C) QSAR Model**3**.

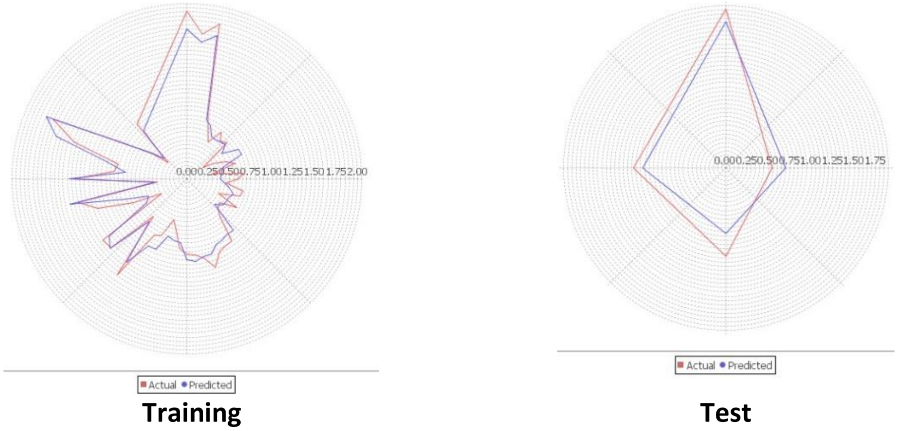

Figure S4: A radar plot representing closeness between the actual and predicted activity of the compounds of training and test set of QSAR model 2.

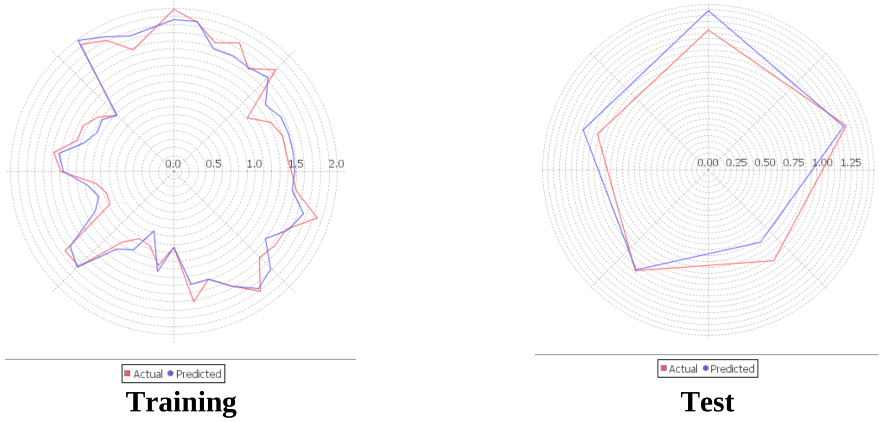

Figure S5: A radar plot representing closeness between the actual and predicted activity of the compounds of training and test set of QSAR model 3.

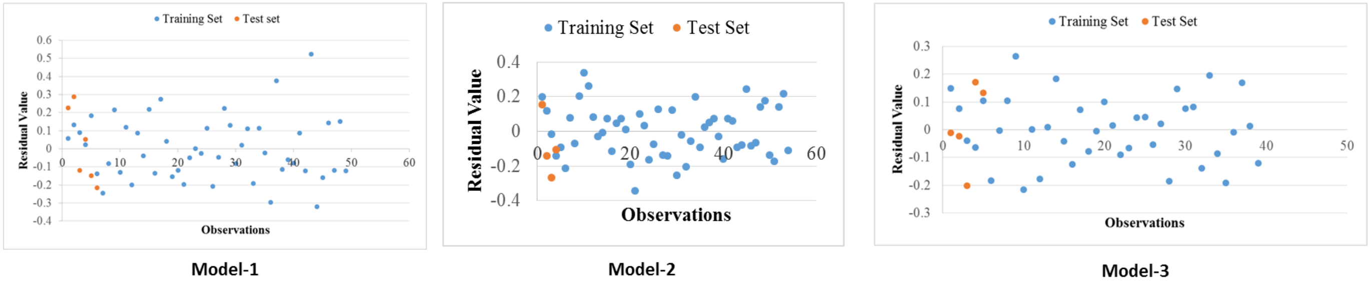

Figure S6: The residual error plot for Model 1, 2and 3.

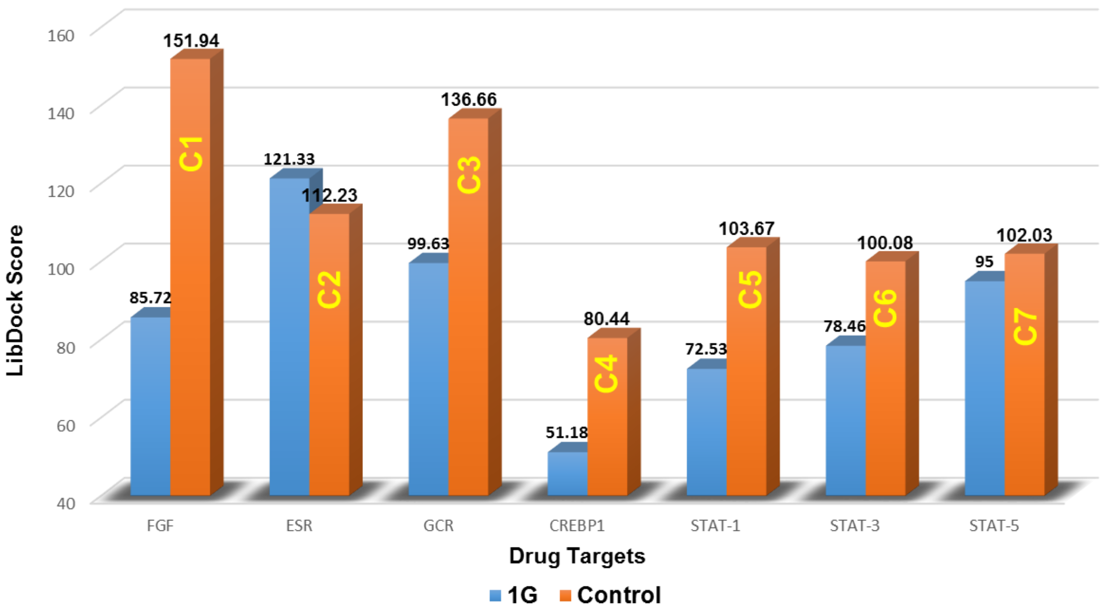

Figure S7: Scoring (LibDock score) of compound 1G in against of different targets identified. The C1 to C5 represent the control compound. Where C1 (66T/CID: 51039095), C2 (ETC/CID: 4474781), C3 (LSJ/CID: 72710581), C4 (XZ8/CID: 6937521), C5 (PTR/CID: 30819), C6 (CID: 160254), and C7 (CID: 2726045)

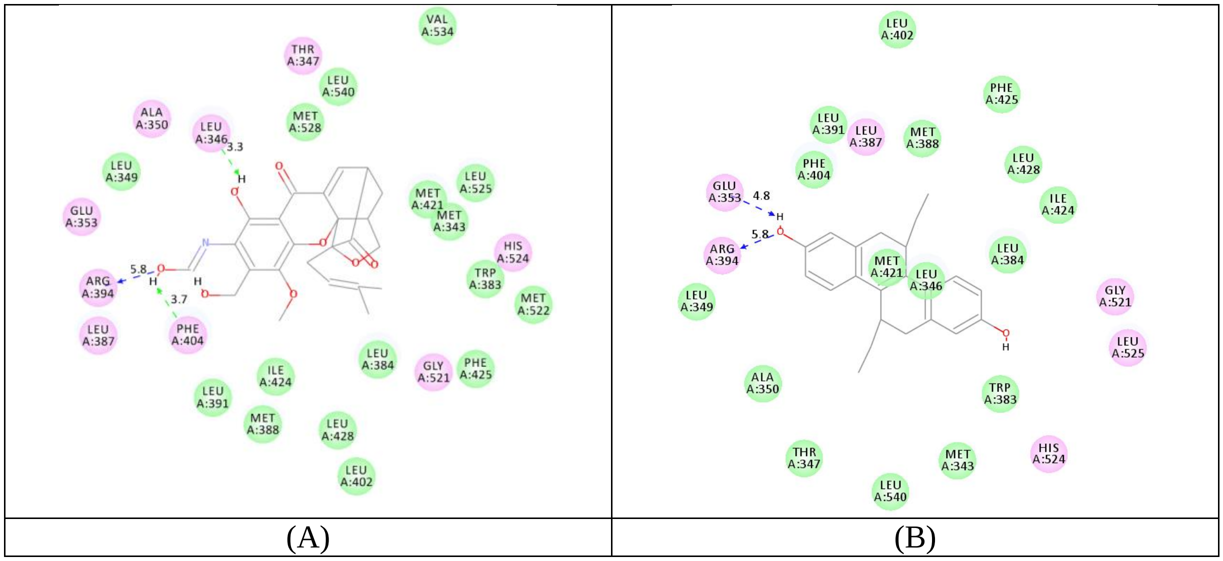

(A) (B)

Figure S8: Two dimensional diagrams illustrating protein–ligand interactions: (A) compound 1G; (B) control compound (ETC). Broken arrow represent the hydrogen bonding with length in Å.

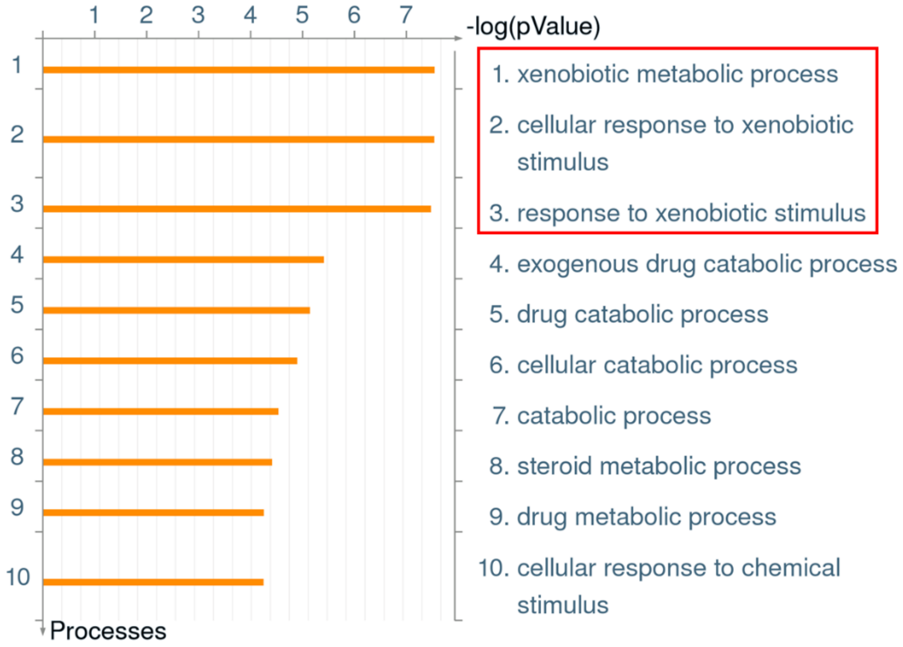

Figure S9: GO process network, shortlisted on the basis of their P value. (Bar length reflects the significance and equals to the negative logarithm of enrichment p-value).

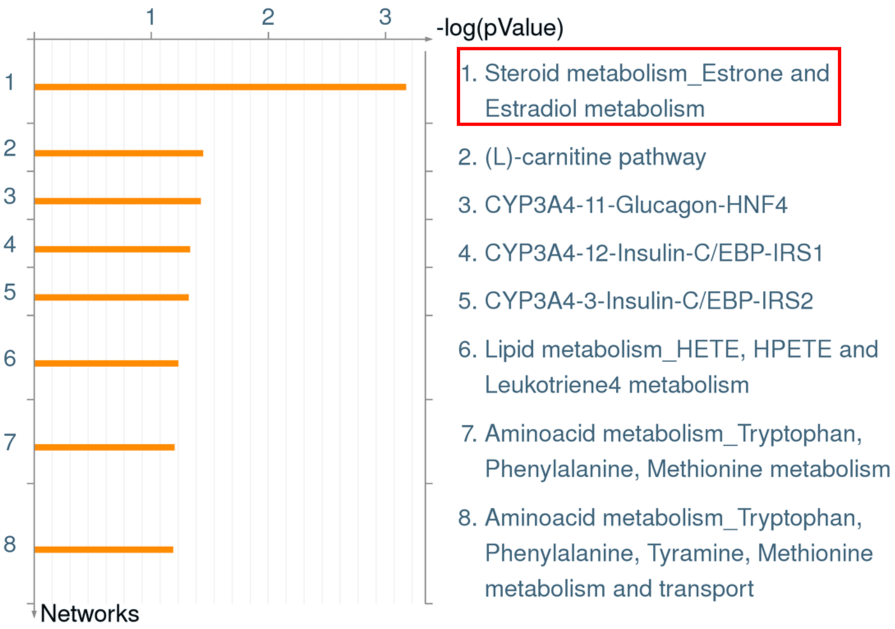

Figure 10: Metabolic Networks of steroid metabolism shortlisted on the basis of their P value. (Bar length reflects the significance and equals to the negative logarithm of enrichment p-value).

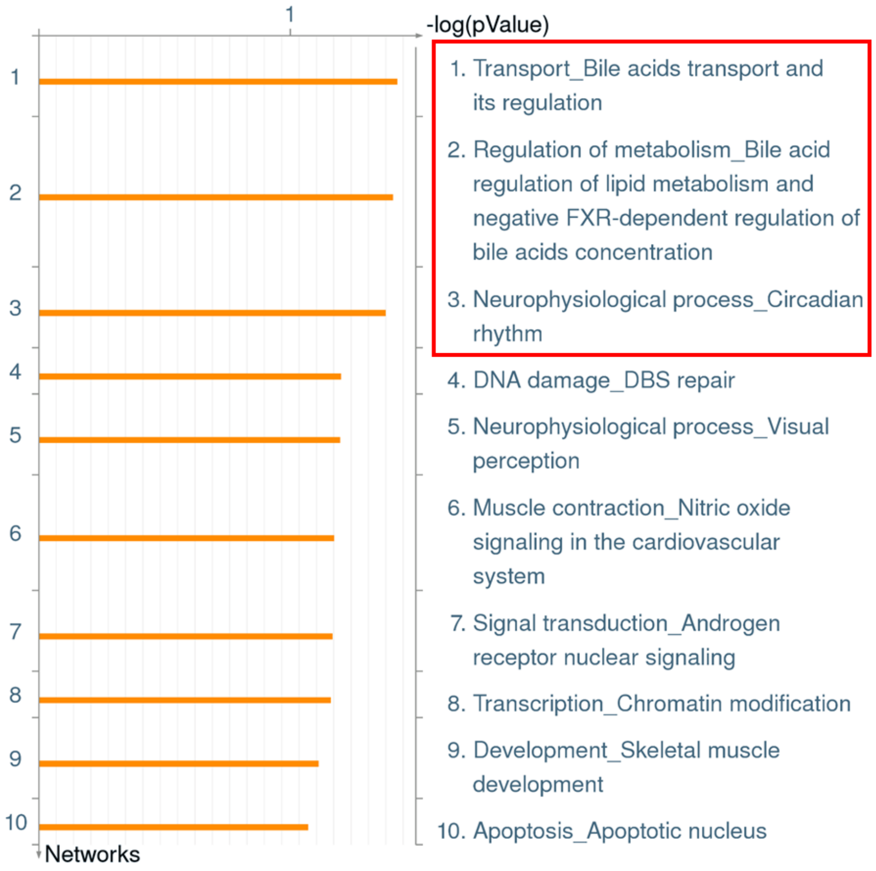

Figure S11: Process network shortlisted on the basis of their P value. (Bar length reflects the significance and equals to the negative logarithm of enrichment p-value).

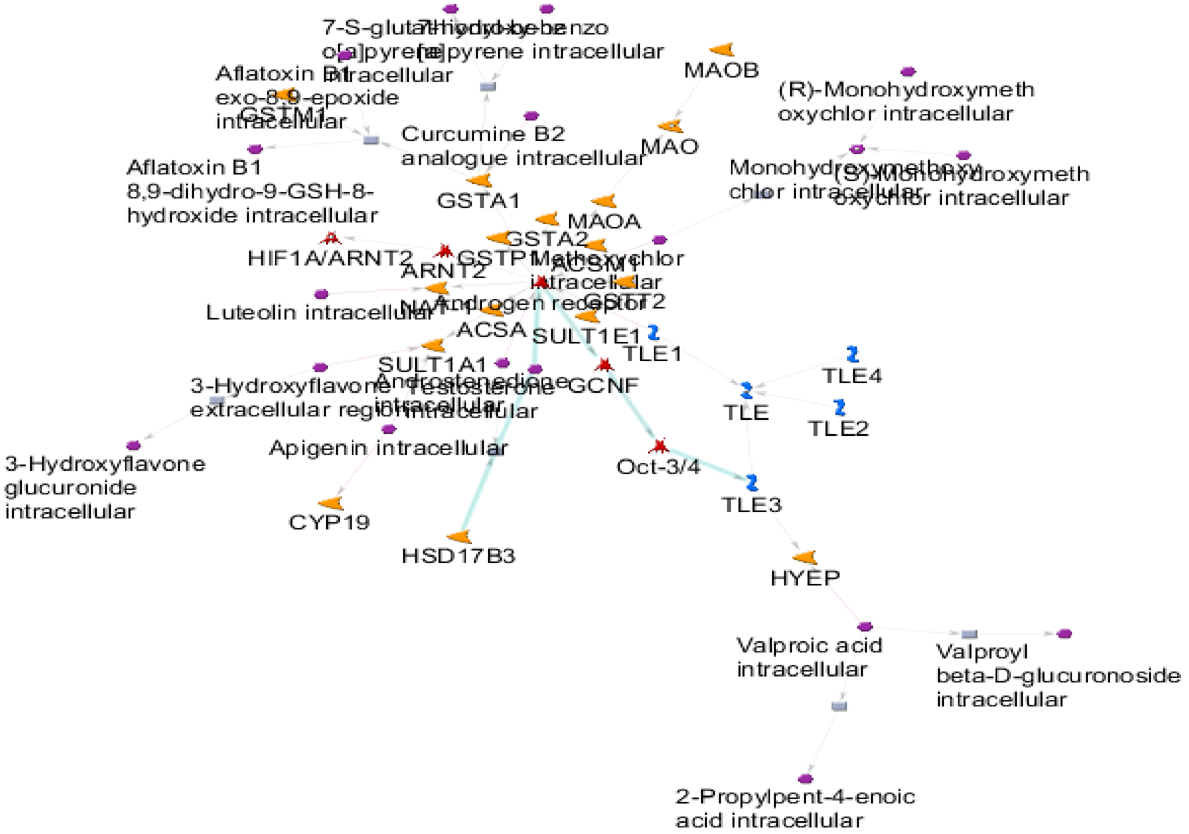

Figure S12. The top scored (by the number of pathways) network from active experiments. Thick cyan lines indicate the fragments of canonical pathways. Up-regulated genes are marked with red circles; down-regulated with blue circles.

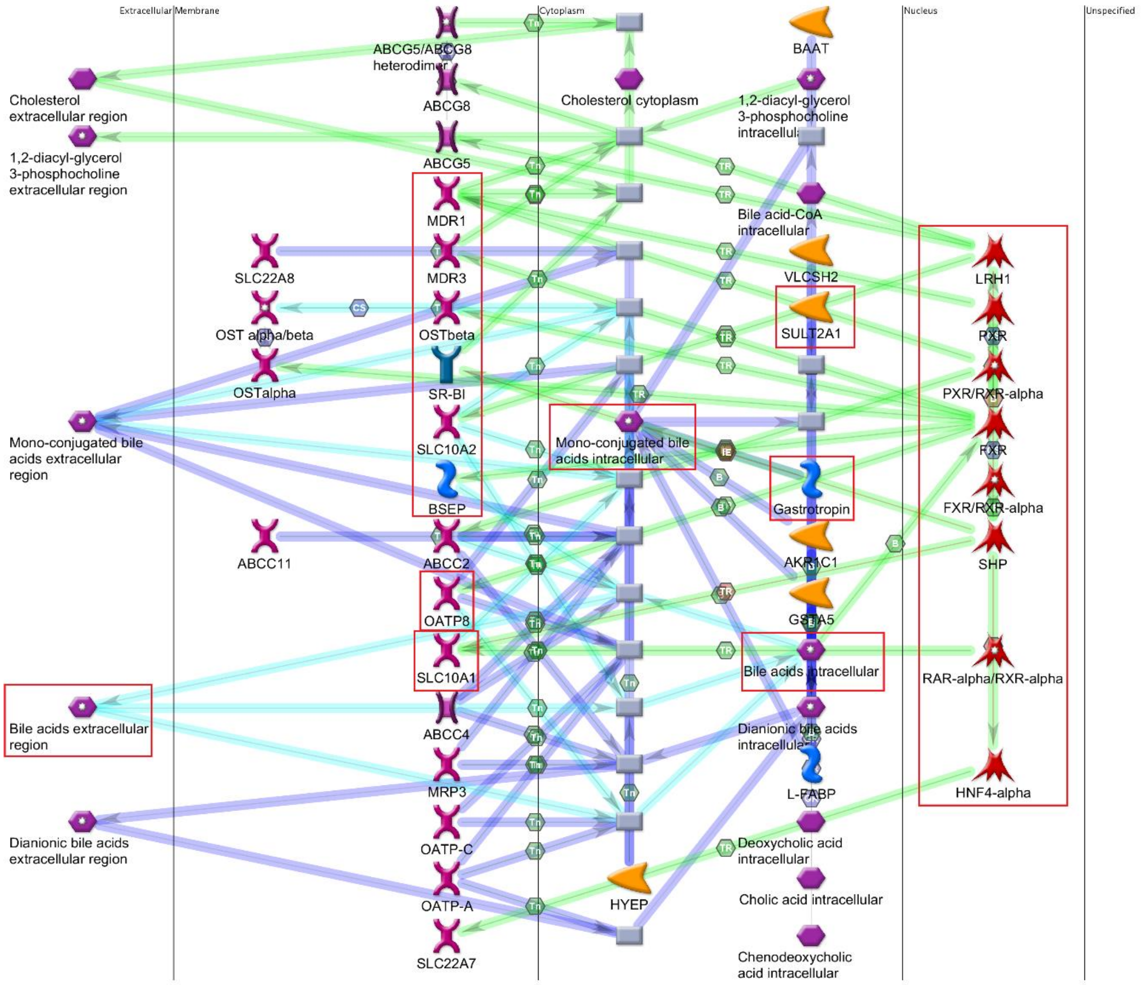

Figure S13: Bile acids transport and its key regulatory proteins and transporters (red encircle).

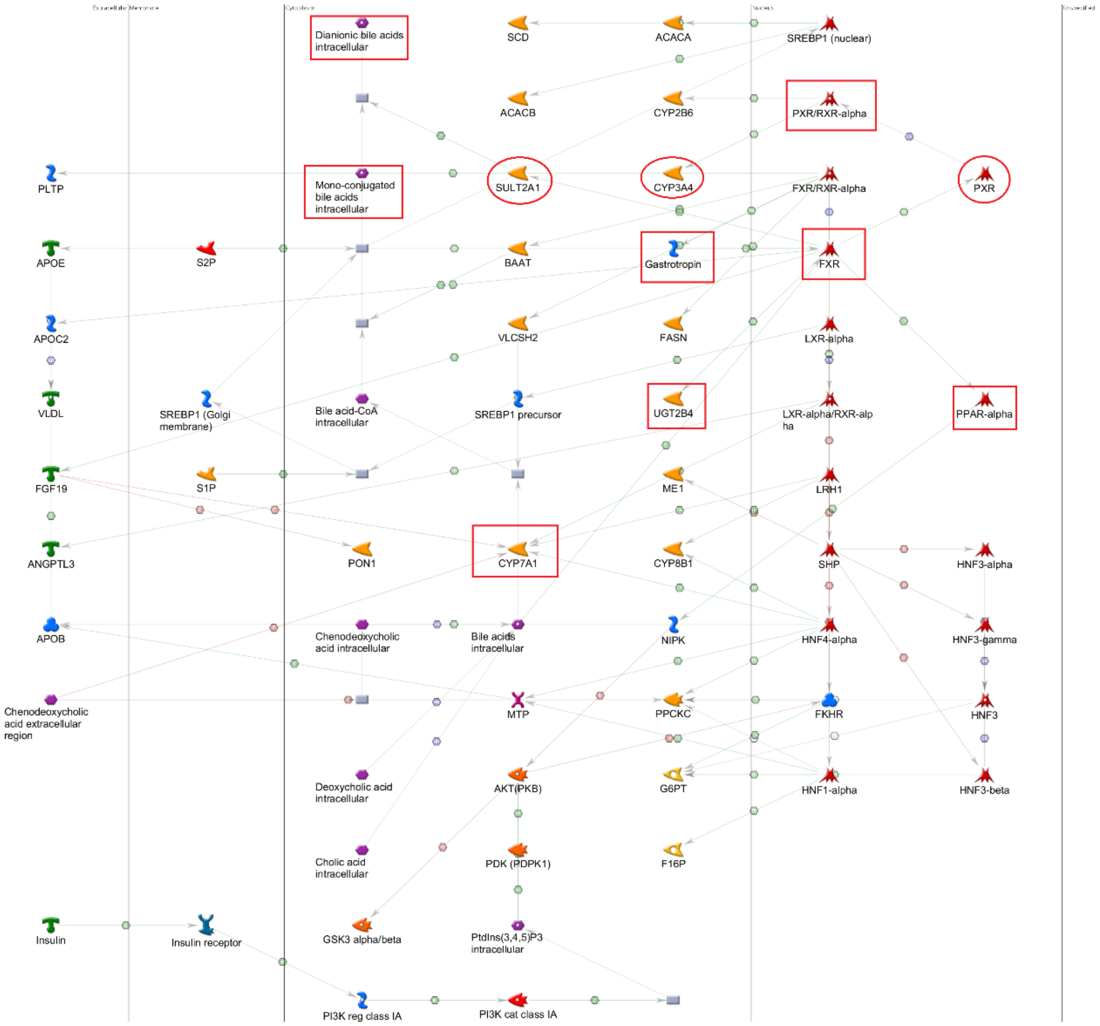

Figure S14: Regulation of metabolism by Bile acid regulation of lipid metabolism and negative FXR-dependent regulation of bile acids concentration. The key protein and transporters are encircled with red.

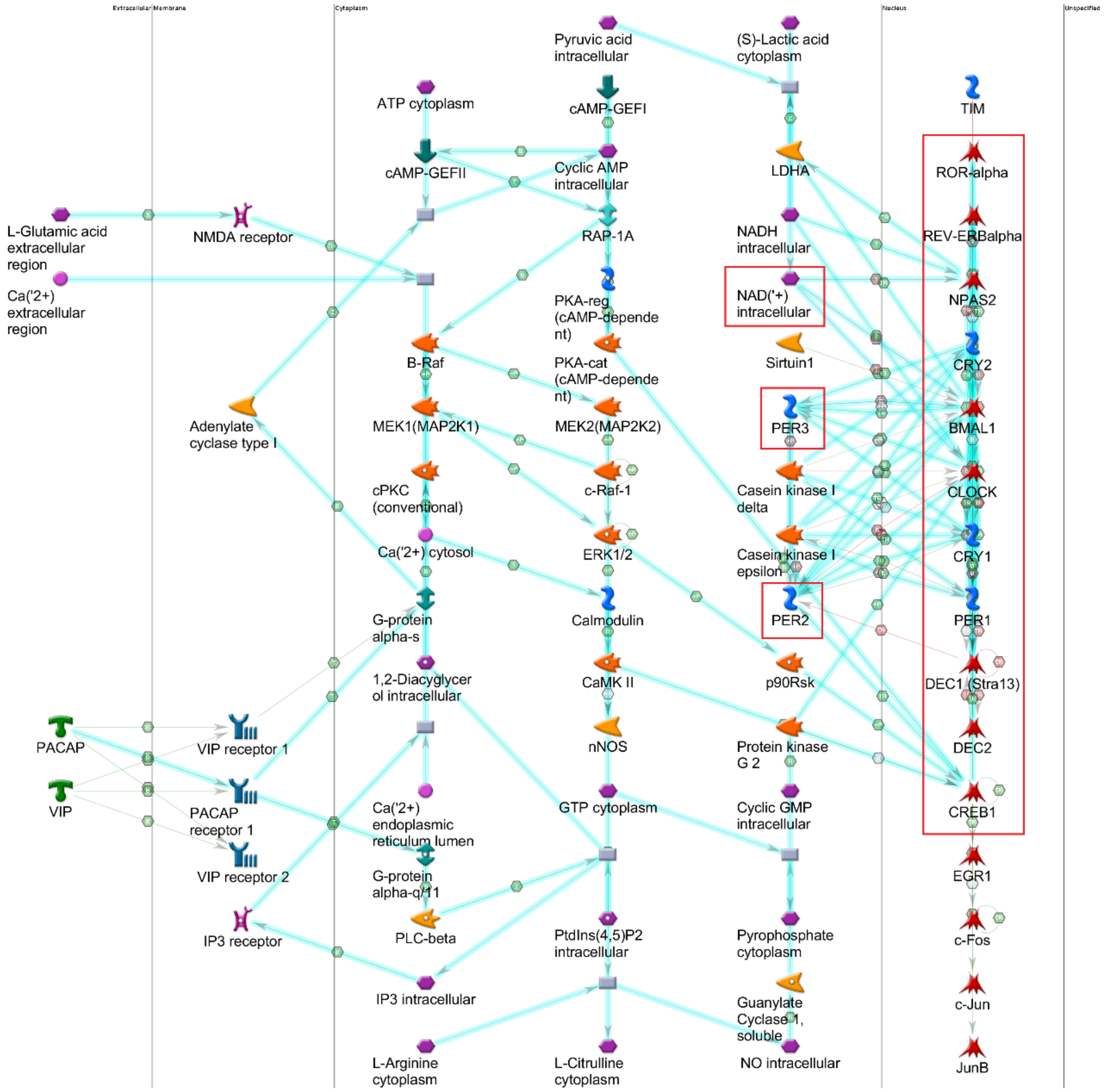

Figure S15: Circadian rhythm regulated proteins and transporters (red encircle).

Table S1: Comparison of experimental and predicted activities of training data set molecules based on QSAR model for A549 cell line.

Serial Number	Compound-ID	Compound Structure	Experimental Activity Log1_IC50	Delta EpsilonC	MMFF_29	SssssCcount	T_2_2_1	SdsCHE-index	Prediction	Error factor**	Applicability Domain
1.	Gar-23	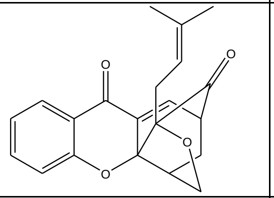	1.56	-0.063	0	2	12	3.883	1.62	0.06	0
2.	Gar-30	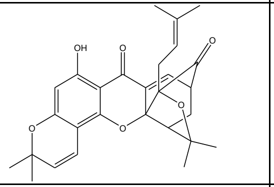	0.5	-0.069	1	4	14	7.545	0.63	0.13	0
3.	Gar-33	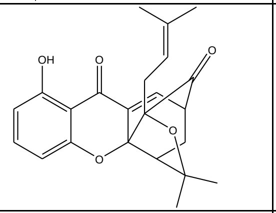	-0.15	-0.07	1	3	12	3.761	-0.06	0.21	0
4.	Gar-34	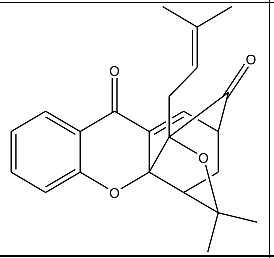	0.29	-0.057	0	3	12	3.903	0.31	0.02	0
5.	Gar-36	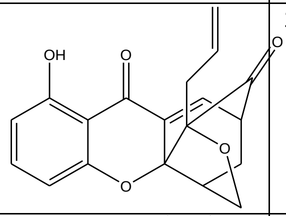	1.27	-0.086	1	2	12	3.381	1.4	0.13	0
6.	Gar-46	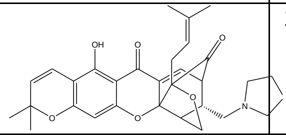	1.85	-0.069	1	3	14	7.539	1.71	-0.14	0
7.	Gar-47	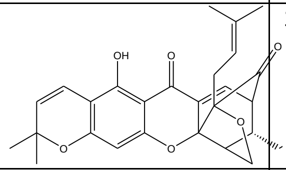	1.85	-0.066	1	3	14	7.555	1.60	-0.25	0
8.	Gar-50	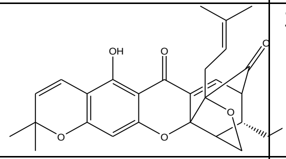	1.56	-0.063	1	3	14	7.539	1.48	-0.08	0
9.	Gar-51	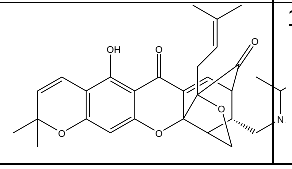	1.25	-0.063	1	3	14	7.515	1.46	0.21	0

10.	Gar-52	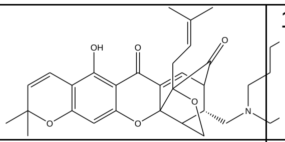	1.47	-0.059	1	3	14	7.572	1.34	-0.13	0
11.	Gar-61	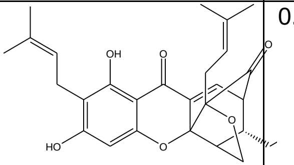	0.84	-0.065	2	2	13	5.717	0.95	0.11	0
12.	Gar-62	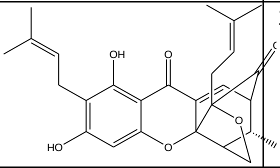	1.56	-0.076	2	2	13	5.661	1.36	-0.2	0
13.	Gar-64	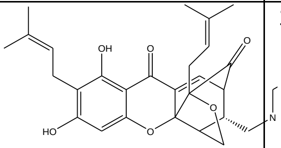	1.02	-0.069	2	2	13	5.703	1.10	0.08	0
14.	Gar-65	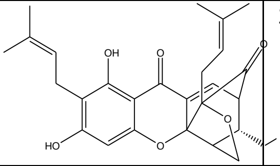	1.24	-0.074	2	2	14	5.597	1.20	-0.04	0
15.	Gar-66	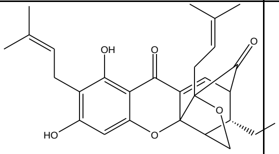	1.22	-0.085	2	2	16	5.408	1.43	0.21	0
16.	Gar-67	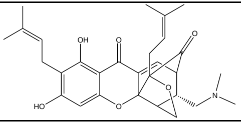	1.24	-0.07	2	2	13	5.622	1.11	-0.13	0
17.	Gar-70	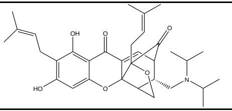	0.51	-0.061	2	2	13	5.683	0.78	0.27	0
18.	Gar-73	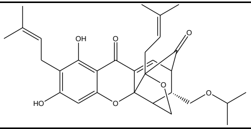	1.11	-0.072	2	2	13	5.558	1.15	0.04	0
19.	Gar-74	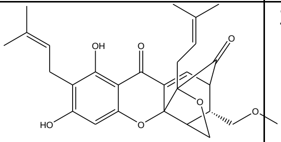	1.76	-0.085	2	2	13	5.455	1.61	-0.15	0
20.	Gar-75	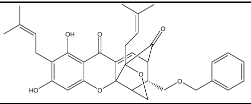	0.71	-0.065	2	2	19	5.563	0.59	-0.12	0
21.	Gar-77	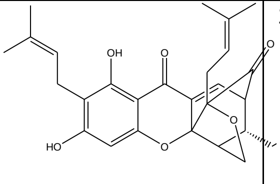	1.71	-0.085	2	2	14	5.366	1.51	-0.2	0
22.	Gar-79	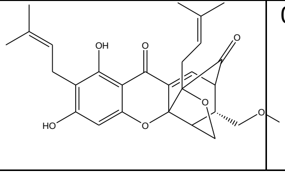	0.78	-0.072	2	2	20	5.393	0.73	-0.05	0

23.	Gar-81	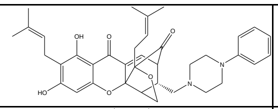	0.51	-0.061	2	2	19	5.711	0.51	0.0	0
24.	Gar-82	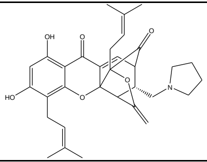	0.92	-0.065	2	2	14	5.685	0.90	-0.02	0
25.	Gar-83	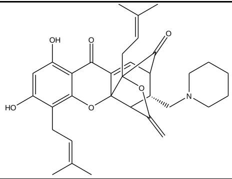	0.71	-0.063	2	2	14	5.7	0.82	0.11	0
26.	Gar-85	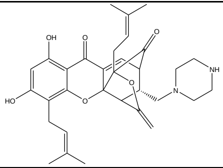	1.25	-0.069	2	2	14	5.67	1.04	-0.21	0
27.	Gar-86	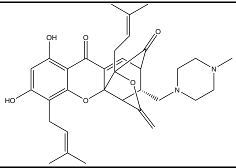	1.02	-0.067	2	2	14	5.685	0.97	-0.05	0
28.	Gar-87	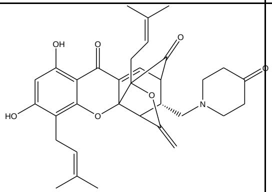	0.8	-0.071	2	2	15	5.573	1.02	0.22	0
29.	Gar-89	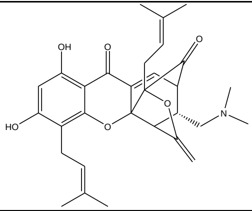	0.8	-0.067	2	2	14	5.599	0.93	0.13	0
30.	Gar-90	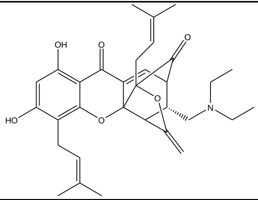	0.88	-0.063	2	2	14	5.648	0.80	-0.08	0
31.	Gar-91	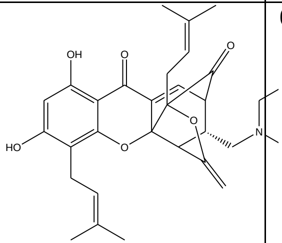	0.64	-0.059	2	2	14	5.685	0.66	0.02	0
32.	Gar-92	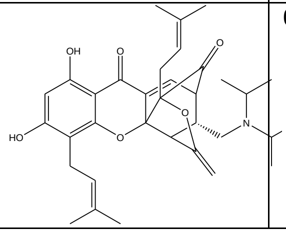	0.54	-0.059	2	2	14	5.664	0.65	0.11	0

33.	Gar-93	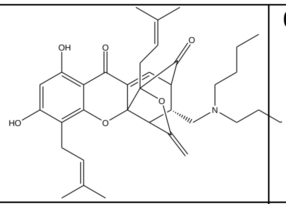	0.75	-0.056	2	2	14	5.715	0.56	-0.19	0
34.	Gar-95	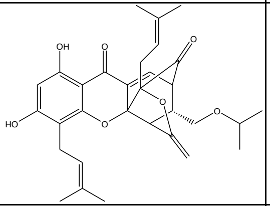	0.9	-0.07	2	2	14	5.533	1.01	0.11	0
35.	Gar-96	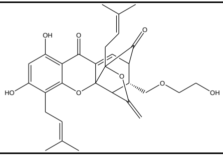	1.45	-0.082	2	2	14	5.424	1.43	-0.02	0
36.	Gar-98	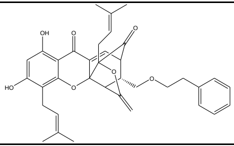	0.68	-0.061	2	2	20	5.554	0.38	-0.3	0
37.	Gar-102	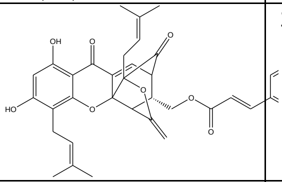	1.48	-0.068	2	2	24	8.311	1.85	0.37	0
38.	Gar-103	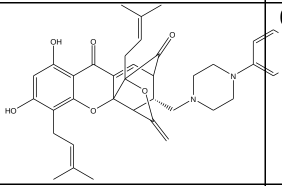	0.49	-0.059	2	2	20	5.693	0.34	-0.15	0
39.	Gar-108	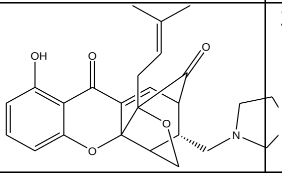	1.1	-0.069	1	2	12	3.876	1.04	-0.06	0
40.	Gar-109	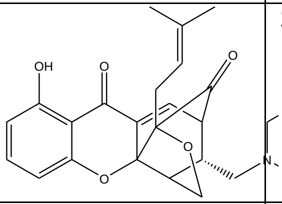	1.01	-0.066	1	2	12	3.888	0.93	-0.08	0
41.	Gar-114	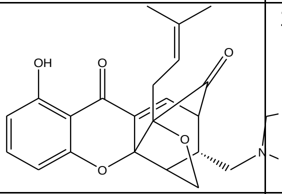	1.58	-0.092	1	2	15	3.627	1.66	0.08	0
42.	Gar-117	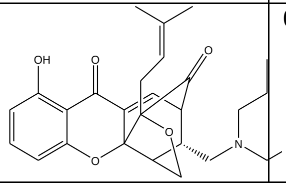	0.89	-0.062	1	2	12	3.876	0.77	-0.12	0

43.	Gar-119	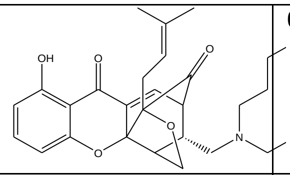	0.1	-0.058	1	2	12	3.9	0.62	0.52	0
44.	Gar-122	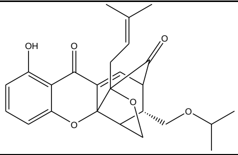	1.57	-0.076	1	2	12	3.749	1.25	-0.32	0
45.	Gar-123	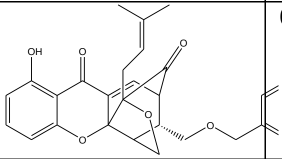	0.77	-0.067	1	2	18	3.753	0.61	-0.16	0
46.	Gar-125	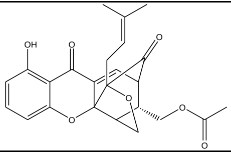	1.56	-0.091	1	2	13	3.584	1.70	0.14	0
47.	Gar-127	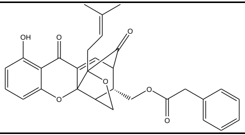	0.88	-0.074	1	2	19	3.607	0.76	-0.12	0
48.	Gar-129	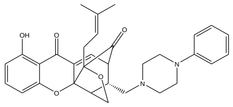	0.33	-0.062	1	2	18	3.883	0.48	0.15	0
49.	Gar-130	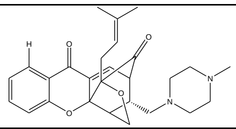	1.73	-0.061	0	2	12	4.018	1.60	-0.13	0

Table S2: Comparison of experimental and predicted activities of training data set molecules based on QSAR model

S.No.	Compound ID	Compound Structure	Log_IC50	H-Acceptor Count	SdsCHE-index	SdssCE-index	T_2_2_2	T_O_O_3	Prediction	Error factor**	AD
1.	HP-21	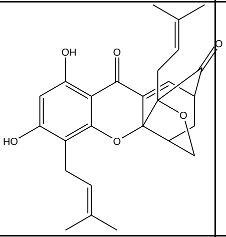	0.5	4	5.61	2.087	14	2	0.69	0.19	0
2.	HP-32	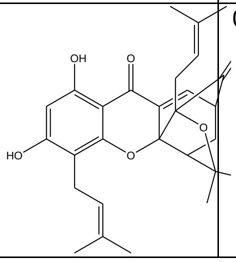	0.61	4	5.634	2.046	14	2	0.73	0.12	0

3.	HP-34	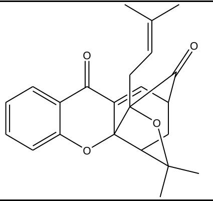	0.83	2	3.903	1.766	13	2	0.81	-0.02	0
4.	HP-46	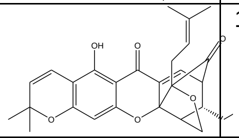	1.85	3	7.539	1.274	16	2	1.71	-0.14	0
5.	HP-49	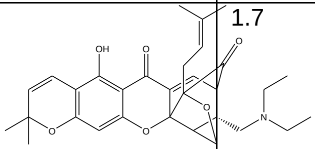	1.7	4	7.497	1.217	16	2	1.61	-0.09	0
6.	HP-54	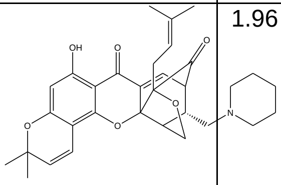	1.96	3	7.723	1.28	16	2	1.75	-0.21	0
7.	HP-61	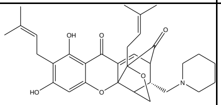	0.6	4	5.717	2.156	14	2	0.67	0.07	0
8.	HP-62	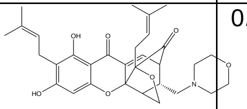	0.8	4	5.661	2.059	14	2	0.73	-0.07	0
9.	HP-63	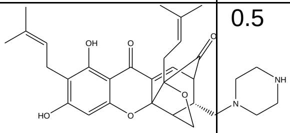	0.5	4	5.689	2.107	14	2	0.70	0.2	0
10.	HP-64	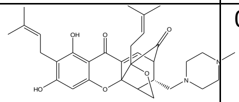	0.36	4	5.703	2.118	14	2	0.70	0.34	0
11.	HP-65	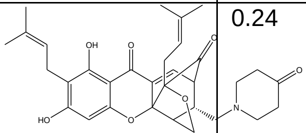	0.24	5	5.597	2.189	14	2	0.50	0.26	0
12.	HP-66	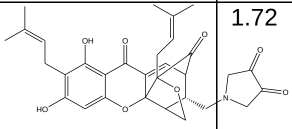	1.72	6	5.408	0.709	16	3	1.80	0.08	0
13.	HP-67	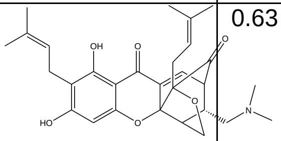	0.63	5	5.622	2.049	14	2	0.60	-0.03	0
14.	HP-68	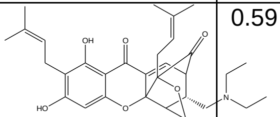	0.59	5	5.668	2.087	14	2	0.59	0.0	0

15.	HP-69	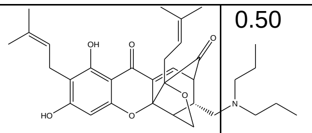	0.50	5	5.703	2.115	14	2	0.57	0.07	0
16.	HP-71	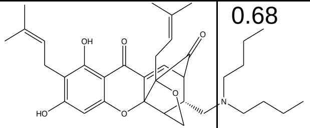	0.68	5	5.731	2.138	14	2	0.57	-0.11	0
17.	HP-72	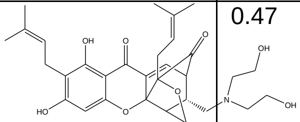	0.47	7	5.476	1.725	14	2	0.51	0.04	0
18.	HP-73	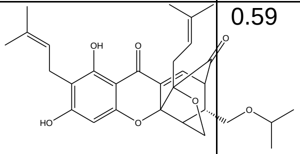	0.59	5	5.558	1.927	14	2	0.66	0.07	0
19.	HP-74	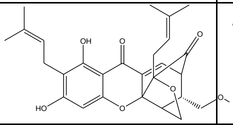	1.20	6	5.455	1.753	14	3	1.21	0.01	0
20.	HP-75	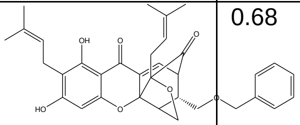	0.68	5	5.563	1.884	20	2	0.49	-0.19	0
21.	HP-79	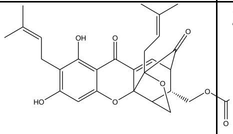	1.1	6	5.393	1.152	21	2	0.76	-0.34	0
22.	HP-80	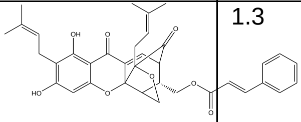	1.3	6	8.376	0.978	25	2	1.40	0.1	0
23.	HP-81	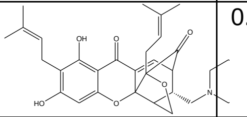	0.49	4	5.711	2.082	20	2	0.52	0.03	0
24.	HP-82	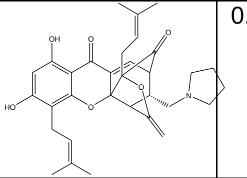	0.67	4	5.685	2.414	14	2	0.51	-0.16	0
25.	HP-84	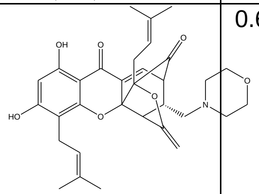	0.65	4	5.64	2.293	14	2	0.57	-0.08	0
26.	HP-85	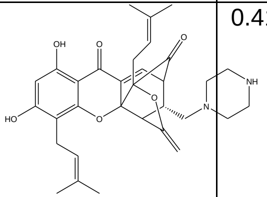	0.41	4	5.67	2.359	14	2	0.53	0.12	0

27.	HP-86	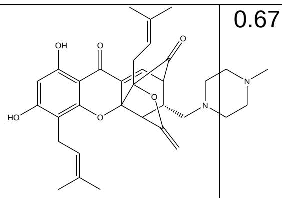	0.67	4	5.685	2.374	14	2	0.53	-0.14	0
28.	HP-89	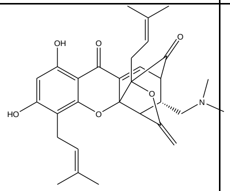	0.59	5	5.599	2.278	14	2	0.45	-0.14	0
29.	HP-90	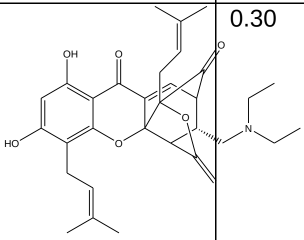	0.30	5	5.648	2.331	14	2	0.42	0.12	0
30.	HP-91	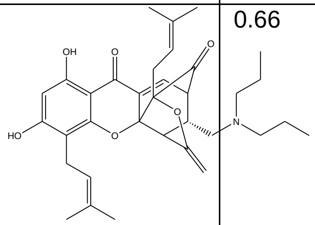	0.66	5	5.685	2.37	14	2	0.41	-0.25	0
31.	HP-92	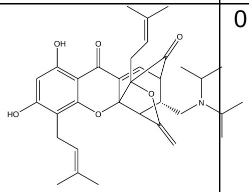	0.46	5	5.664	2.312	14	2	0.44	-0.02	0
32.	HP-93	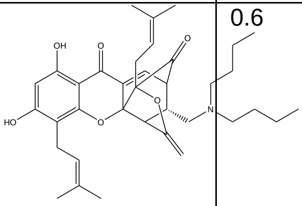	0.6	5	5.715	2.4	14	2	0.40	-0.2	0
33.	HP-94	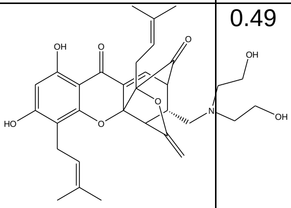	0.49	7	5.447	1.845	14	2	0.44	-0.05	0
34.	HP-95	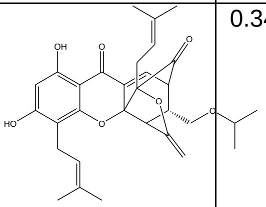	0.34	5	5.533	2.108	14	2	0.54	0.2	0

35.	HP-96	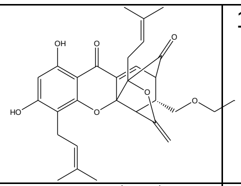	1.22	6	5.424	1.874	14	3	1.13	-0.09	0
36.	HP-97	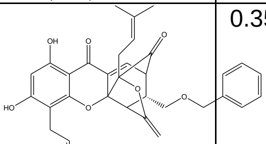	0.35	5	5.537	2.051	20	2	0.37	0.02	0
37.	HP-102	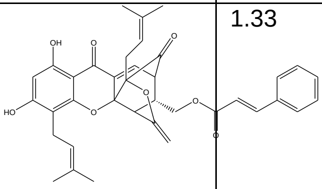	1.33	6	8.311	0.986	25	2	1.38	0.05	0
38.	HP-103	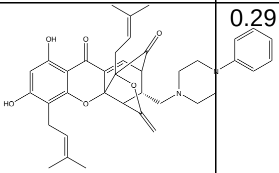	0.29	4	5.693	2.327	20	2	0.36	0.07	0
39.	HP-108	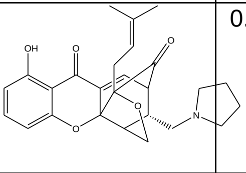	0.95	3	3.876	1.39	13	2	0.92	-0.03	0
40.	HP-109	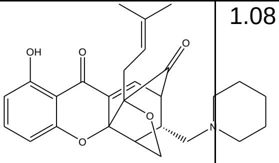	1.08	3	3.888	1.398	13	2	0.92	-0.16	0
41.	HP-110	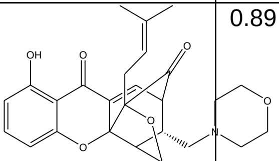	0.89	3	3.841	1.308	13	2	0.97	0.08	0
42.	HP-111	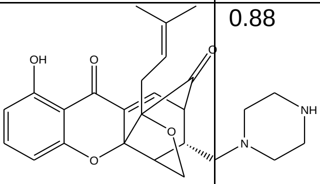	0.88	3	3.864	1.353	13	2	0.94	0.06	0
43.	HP-115	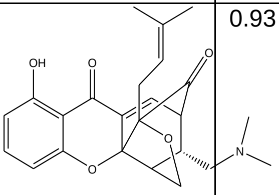	0.93	4	3.806	1.298	13	2	0.84	-0.09	0

44.	HP-116	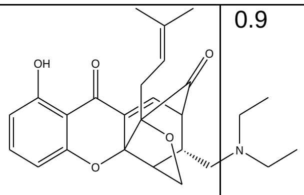	0.9	4	3.846	1.334	13	2	0.82	-0.08	0
45.	HP-117	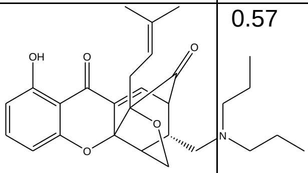	0.57	4	3.876	1.36	13	2	0.81	0.24	0
46.	HP-119	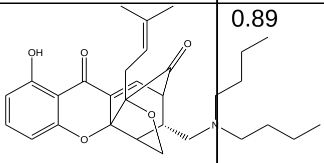	0.89	4	3.9	1.381	13	2	0.81	-0.08	0
47.	HP-120	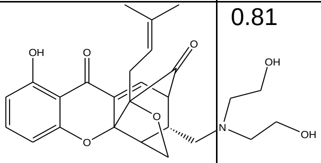	0.81	6	3.685	1.001	13	2	0.75	-0.06	0
48.	HP-121	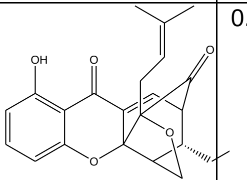	0.76	4	3.722	1.171	13	2	0.90	0.14	0
49.	HP-122	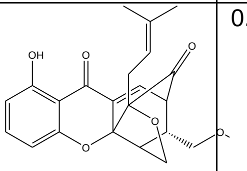	0.72	4	3.749	1.183	13	2	0.89	0.17	0
50.	HP-123	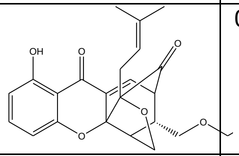	0.86	4	3.753	1.144	19	2	0.72	-0.14	0
51.	HP-125	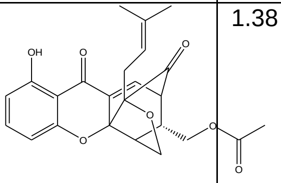	1.38	5	3.584	0.446	13	2	1.21	-0.17	0
52.	HP-127	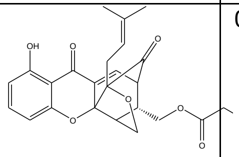	0.82	5	3.607	0.468	20	2	0.96	0.14	0
53.	HP-128	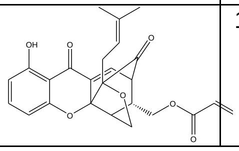	1.39	5	6.626	0.297	24	2	1.60	0.21	0

54.	HP-129	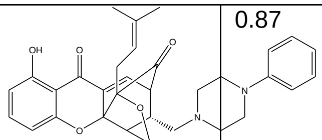	0.87	3	3.883	1.331	19	2	0.76	-0.11	0

Table S3: Comparison of experimental and predicted activities of training data set molecules based on QSAR model

S.No.	Compound ID	Compound Structure	Log10_IC50	SssssCE-index	T_T_N_4	T_O_O_3	Prediction	Error factor**	AD
1.	U-19	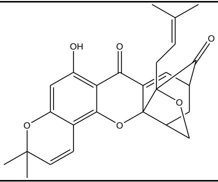	0.92	-2.995	0	2	1.07	0.15	0
2.	U-21	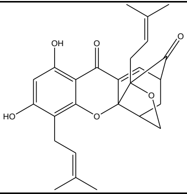	1.16	-2.543	0	2	1.23	0.07	0
3.	U-22	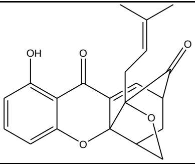	1.38	-2.261	0	2	1.34	-0.04	0
4.	U-23	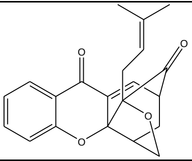	1.48	-2.054	0	2	1.42	-0.06	0
5.	U-28	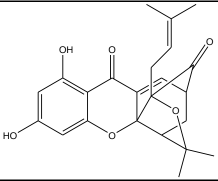	0.86	-3.283	0	2	0.96	0.1	0
6.	U-30	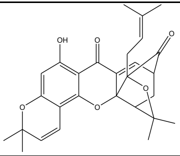	0.95	-3.817	0	2	0.78	-0.17	0
7.	U-32	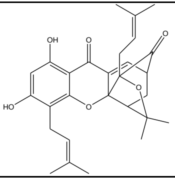	0.93	-3.387	0	2	0.93	0.0	0
8.	U-33	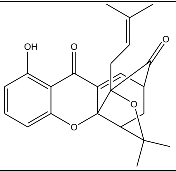	0.96	-3.01	0	2	1.06	0.1	0
9.	U-55	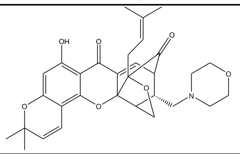	1.12	-3.172	4	2	1.38	0.26	0

10.	U-57	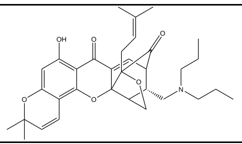	1.61	-3.148	4	2	1.39	-0.22	0
11.	U-58	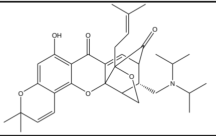	1.38	-3.184	4	2	1.38	0.0	0
12.	U-60	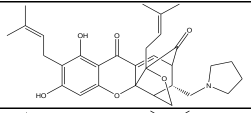	1.77	-2.607	4	2	1.59	-1.98	0
13.	U-62	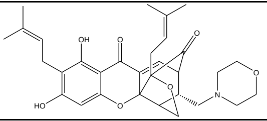	1.56	-2.67	4	2	1.56	0.0	0
14.	U-63	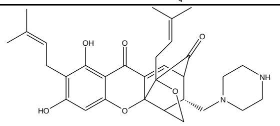	1.49	-2.642	5	2	1.67	0.18	0
15.	U-64	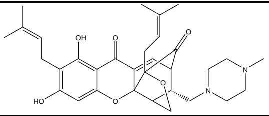	1.81	-2.645	6	2	1.77	-0.04	0
16.	U-65	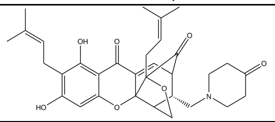	1.76	-2.747	5	2	1.64	-0.12	0
17.	U-66	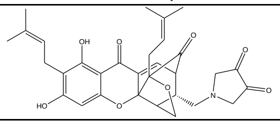	1.92	-2.941	4	3	1.99	0.07	0
18.	U-67	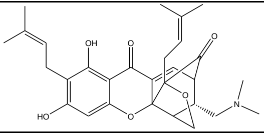	1.66	-2.637	4	2	1.58	-0.08	0
19.	U-70	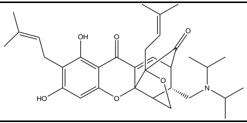	1.57	-2.681	4	2	1.57	0.0	0
20.	U-73	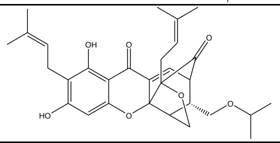	1.07	-2.725	0	2	1.17	0.1	0
21.	U-74	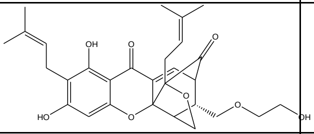	1.64	-2.821	0	3	1.65	0.01	0
22.	U-75	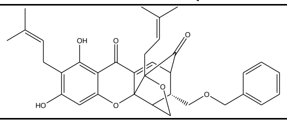	1.24	-2.783	0	2	1.15	-1.09	0
23.	U-79	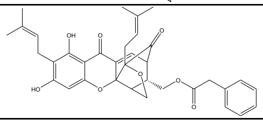	1.14	-2.987	0	2	1.07	-0.07	0
24.	U-81	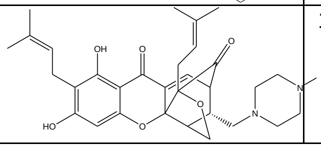	1.80	-2.697	7	2	1.84	0.04	0

25.	U-83	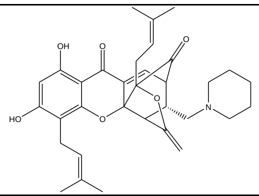	1.44	-2.895	4	2	1.48	0.04	0
26.	U-84	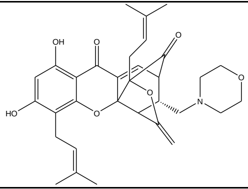	1.52	-2.951	4	2	1.47	-0.05	0
27.	U-85	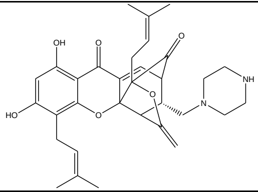	1.55	-2.923	5	2	1.57	0.02	0
28.	U-86	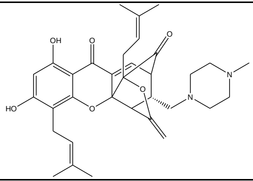	1.85	-2.926	6	2	1.67	-0.18	0
29.	U-89	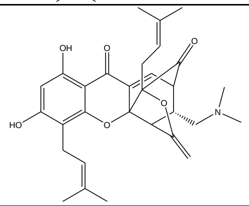	1.33	-2.918	4	2	1.47	0.14	0
30.	U-90	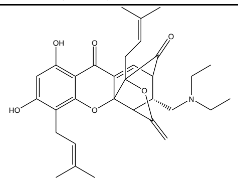	1.40	-2.924	4	2	1.47	0.07	0
31.	U-91	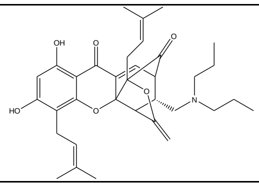	1.39	-2.93	4	2	1.47	0.08	0
32.	U-94	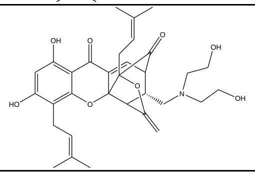	1.53	-3.154	4	2	1.39	-0.14	0
33.	U-95	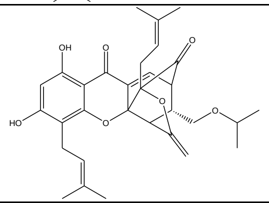	0.87	-3.006	0	2	1.07	0.2	0
34.	U-96	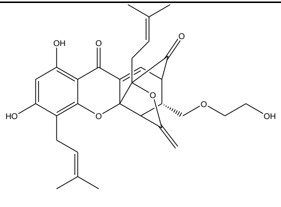	1.64	-3.102	0	3	1.55	-0.09	0

35.	U-98	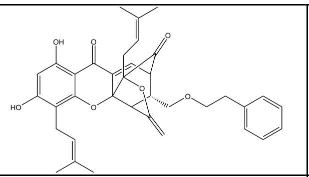	1.24	-3.053	0	2	1.05	-0.19	0
36.	U-101	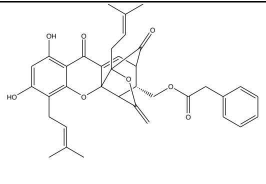	0.98	-3.268	0	2	0.97	-0.01	0
37.	U-103	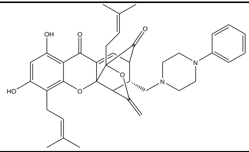	1.57	-2.978	7	2	1.74	0.17	0
38.	U-112	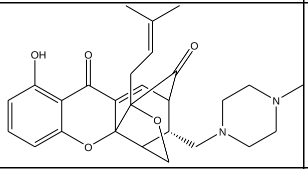	1.85	-2.388	6	2	1.86	0.01	0
39.	U-119	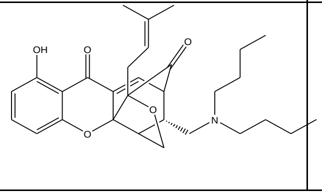	1.98	-2.398	6	2	1.86	-0.12	0

Table S4: Comparison of experimental and predicted activities of test data set molecules based on QSAR model

S.No.	Compound ID	Compound Structure	Experimental Activity Log1_IC50	Delta EpsilonC	MMFF_29	SssssC count	T_2_2_1	SdsCHE-index	Prediction	**Error factor** **	**AD**
1.	Gar-29	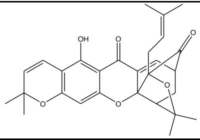	0.33	-0.069	1	4	14	7.392	0.55	0.22	0
2.	Gar-54	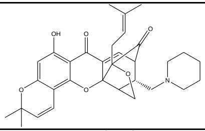	1.4	-0.066	1	3	14	7.723	1.68	0.28	0
3.	Gar-78	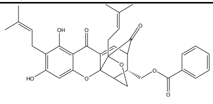	0.87	-0.074	2	2	21	5.377	0.75	-0.12	0
4.	Gar-97	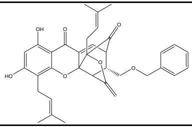	0.4	-0.063	2	2	20	5.537	0.45	0.05	0
5.	Gar-124	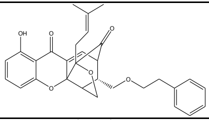	0.65	-0.064	1	2	18	3.766	0.50	-0.15	0
6.	Gar-126	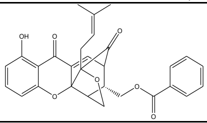	1.04	-0.077	1	2	20	3.594	0.82	-0.22	0

Table S5: Comparison of experimental and predicted activities of test data set molecules based on QSAR model

Serial Number	Compound ID	Compound Structure	Log_IC50	H-Acceptor Count	SdsCHE-index	SdssCE-index	T_2_2_2	T_O_O_3	Prediction	Error factor**	AD[1]
1.	HP-20	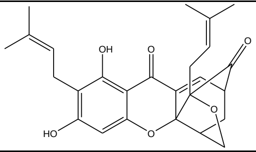	0.54	4	5.551	2.071	14	2	0.69	0.15	0
2.	HP-47	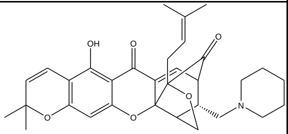	1.85	3	7.555	1.281	16	2	1.71	-0.14	0
3.	HP-78	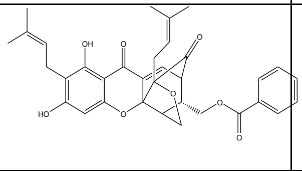	1.03	6	5.377	1.037	23	2	0.76	-0.27	0
4.	HP-126	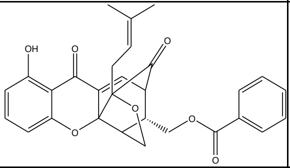	1.07	5	3.594	0.354	22	2	0.96	-0.11	0

Table S6: Comparison of experimental and predicted activities of test data set molecules based on QSAR model

Serial N0	Compound ID	Compound Structure	Log10_ IC_{50}	SssssCE-index	T_T_N_4	T_O_O_3	Prediction	**Error factor****	**AD**
1.	U-18	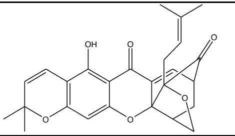	1.09	-2.974	0	2	1.08	-0.01	0
2.	U-20	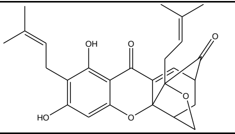	1.27	-2.517	0	2	1.25	-0.02	0
3.	U-29	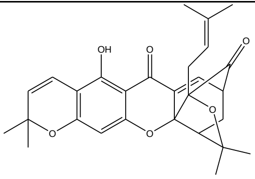	0.98	-3.79	0	2	0.78	-0.2	0
4.	U-50	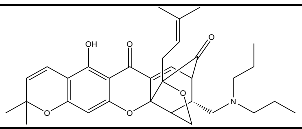	1.23	-3.127	4	2	1.40	0.17	0
5.	U-76	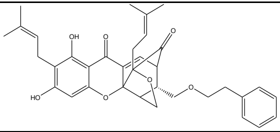	1.02	-2.772	0	2	1.15	0.13	0

**The difference between predicted activity values and experimental activity values is represented as error (ratio between the predicted and experimental activity), with a negative sign if the actual activity is higher than that of the predicted activity.

Table S7: Correlation matrix of the selected descriptors for model 1.

	SssssCcount	SdsCHE-index	DeltaEpsilonC	T_2_2_1	MMFF_29
**SssssCcount**	1	0.463	0.171	-0.156	-0.428
**SdsCHE-index**	0.463	1	0.243	0.268	0.382
**DeltaEpsilonC**	0.171	0.243	1	0.083	-0.086
**T_2_2_1**	-0.156	0.268	0.083	1	0.326
**MMFF_29**	-0.428	0.382	-0.086	0.326	1

Table S8: Correlation matrix of the selected descriptors for model 2.

	H-AcceptorCount	SdsCHE-index	SdssCE-index	T_2_2_2	T_O_O_3
**H-AcceptorCount**	1	0.277	-0.032	0.253	0.342
**SdsCHE-index**	0.277	1	0.206	0.475	0.026
**SdssCE-index**	-0.032	0.206	1	-0.356	-0.104
**T_2_2_2**	0.253	0.475	-0.356	1	-0.054
**T_O_O_3**	0.342	0.026	-0.104	-0.054	1

Table S9: Correlation matrix of the selected descriptors for model 3.

	SssssCE-index	T_T_N_4	T_O_O_3
**SssssCE-index**	1	0.229	-0.069
**T_T_N_4**	0.229	1	-0.171
**T_O_O_3**	-0.069	-0.171	1

Table S10: Synthetic accebility score, structure and predicted activities of query compound screened through QSAR model-1.

S.No.	Structure	IC_{50}	Synthetic accebility score
1D	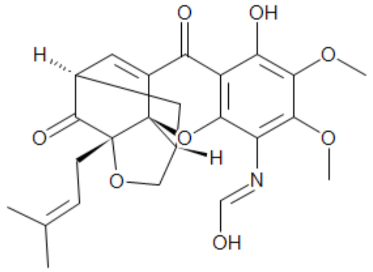	2.04	7.4
1G	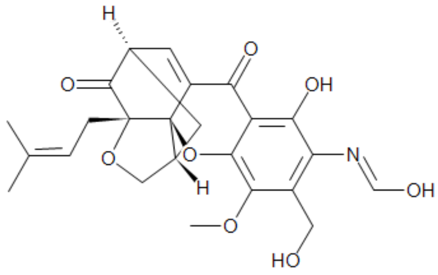	6.32	7.4

1H	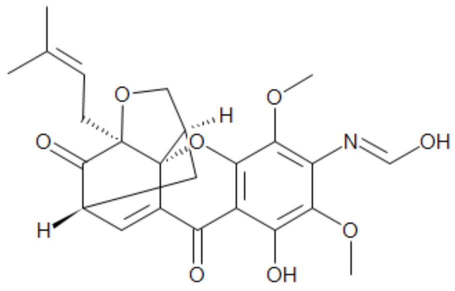	11.12	7.4
2D	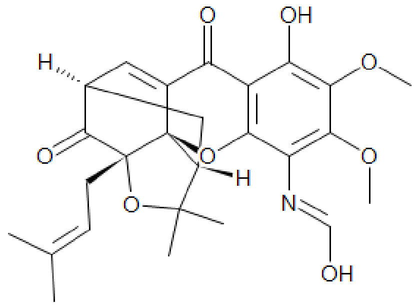	11.25	7.61
2G	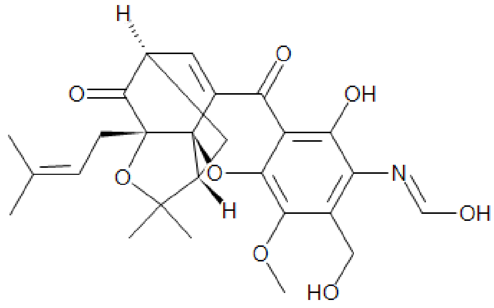	11.35	7.61
3G1	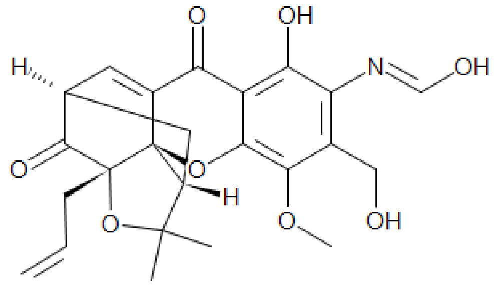	18.15	7.43
3H	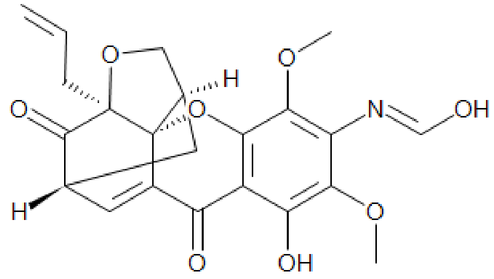	18.49	7.22

3H1	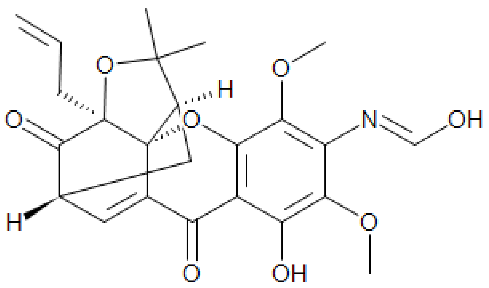	18.15	7.44

Table S11: Synthetic accebility score, structure and predicted activities of query compound screened through QSAR model-2.

**S.No.**	**Structure**	**IC50**	**Synthetic accebility score**
18_4	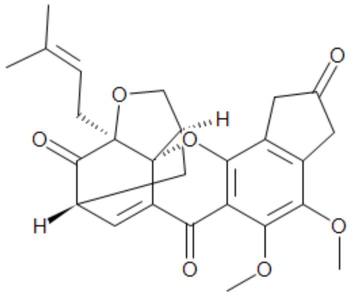	16.6	7.42
18_5	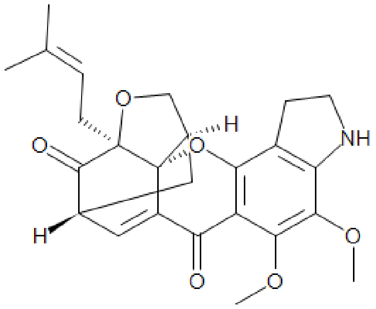	19.8	7.31
18_5DH	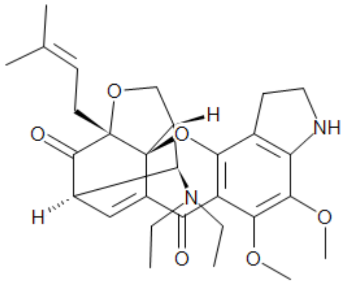	15.9	7.87

18_5FH	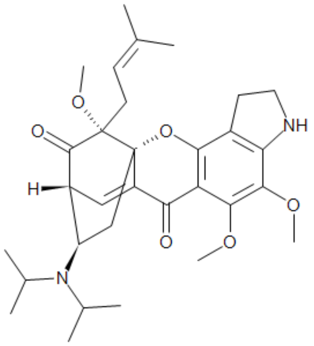	14.4	7.67
18_7ZH	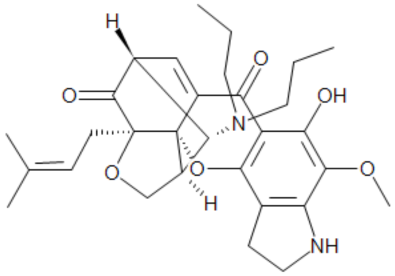		7.87
B-18_4	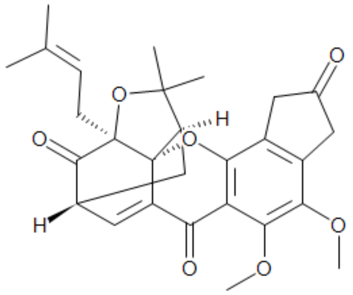	17.8	7.64

Table S12: Synthetic accebility score, structure and predicted activities of query compound screened through QSAR model-3.

**S.No.**	**Structure**	**IC50**	**Synthetic accebility score**
18	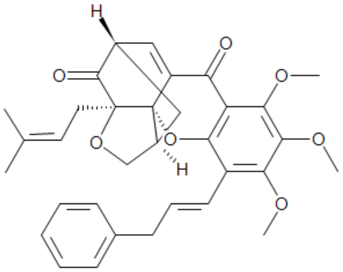	11.96	7.64

1A	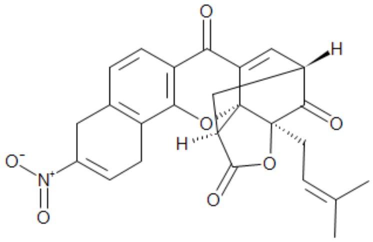	16.48	7.72
2A	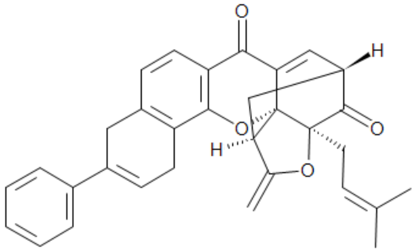	19.18	7.73
3A	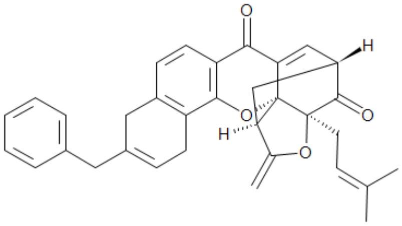	19.31	7.76
5D	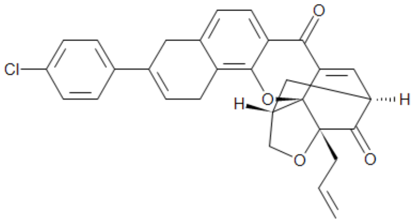	23.22	7.43
6A	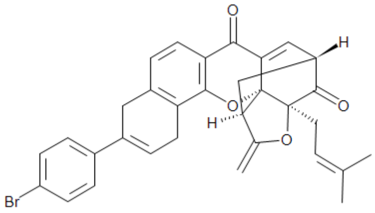	19.27	7.78
7A	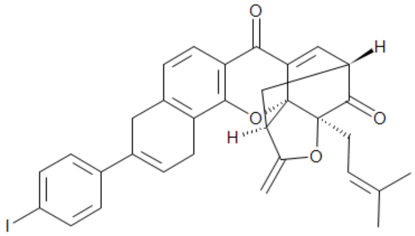	19.31	7.79

Table S13: Bio-physicochemical properties of compound 1G along with control compound Topotecan.

Properties	Compound 1G	Topotecan
DiffCoef	0.58	0.65
MlogP	2.08	1.6
S+logP	3.45	1.9
S+logD	3.45	1.51
S+logHLC	-8.21	-12.51
S+Peff	2.47	0.97
S+MDCK	381.31	190.26
S+Pcornea	67.35	78.05
S+Pskin	24.09	1.9
S+Sw	7.87E-03	4.27E-01
S+pH	7	8.64
S+IS	7.87E-03	3.66E-01
S+SF	1.50E+03	1.41E+02
S+Sp	7.87E-03	9.06E-01
S+FaSSGF	2.11E-03	1.73E+00
S+FaSSIF	1.86E-02	2.34E-01
S+FeSSIF	1.16E-01	3.83E-01
S+SSR	SupSat	SupSat
S+BBB_Filter	High	Low
S+LogBB	-0.02	-0.43
S+PrUnbnd	4.97	17.45
S+Vd	5.33	1.85
S+RBP	0.72	1.04
S+fumic	0.405	0.735
S+Pgp_Substr	Yes (58%)	Yes (65%)
S+Pgp_Inh	Yes (97%)	No (94%)
S+OATP1B1_Inh	Yes (80%)	No (97%)
RuleOf5	1	0
RuleOf5_Code	Mw	

Table S14: Metabolism study of the compound 1G and control compound Topotecan.

Properties	Compound 1G	Topotecan
MET_1A2_Inh	Yes	No
CYP_1A2_Substr	No	No
CYP_1A2_Sites	NonSubstrate	NonSubstrate
MET_1A2_Km	NonSubstrate	NonSubstrate
MET_1A2_Vmax	NonSubstrate	NonSubstrate
MET_1A2_CLint	NonSubstrate	NonSubstrate
CYP_2A6_Substr	No	No
CYP_2A6_Sites	NonSubstrate	NonSubstrate
CYP_2B6_Substr	No	No
CYP_2B6_Sites	NonSubstrate	NonSubstrate
CYP_2C8_Substr	No	No
CYP_2C8_Sites	NonSubstrate	NonSubstrate
MET_2C9_Inh	Yes	No
CYP_2C9_Substr	No	No

CYP_2C9_Sites	NonSubstrate	NonSubstrate
MET_2C9_Km	NonSubstrate	NonSubstrate
MET_2C9_Vmax	NonSubstrate	NonSubstrate
MET_2C9_CLint	NonSubstrate	NonSubstrate
MET_2C19_Inh	Yes	No
CYP_2C19_Substr	No	No
CYP_2C19_Sites	NonSubstrate	NonSubstrate
MET_2C19_Km	NonSubstrate	NonSubstrate
MET_2C19_Vmax	NonSubstrate	NonSubstrate
MET_2C19_CLint	NonSubstrate	NonSubstrate
MET_2D6_Inh	Yes	Yes
CYP_2D6_Substr	No	Yes
CYP_2D6_Sites	NonSubstrate	C31(991); C30(991); C19(744); C26(707)
MET_2D6_Km	NonSubstrate	1.32E+01
MET_2D6_Vmax	NonSubstrate	8.37E+00
MET_2D6_CLint	NonSubstrate	5.07E+00
CYP_2E1_Substr	No	No (98%)
CYP_2E1_Sites	NonSubstrate	NonSubstrate
MET_3A4_Inh	Yes	Yes
CYP_3A4_Substr	Yes	Yes
CYP_3A4_Sites	C13(749); C16(593); C24(516); C21(474)	C31(998); C30(998); C26(897); C19(745)
MET_3A4_Km	1.89E+01	5.74E+01
MET_3A4_Vmax	1.40E+01	1.80E+00
MET_3A4_CLint	8.25E+01	3.48E+00
MET_3A4_HLM_Km	2.36E+02	5.97E+01
MET_3A4_HLM_Vmax	6.70E-01	8.04E-01
MET_3A4_HLM_CLint	2.84E+00	1.35E+01
MET_HLM_Total_CLint	1.82E+01	1.64E+01
MET_3A4_I_mid	Yes	Yes
MET_3A4_I_tes	Yes	No
MET_3A4_Ki_mid	0.689	14.938
MET_3A4_Ki_tes	94.977	61.559
MET_UGT1A1	NoSites	Yes
MET_UGT1A3	NoSites	No
MET_UGT1A4	NoSites	No
MET_UGT1A6	NoSites	No
MET_UGT1A8	NoSites	Yes
MET_UGT1A9	NoSites	No
MET_UGT1A10	NoSites	Yes
MET_UGT2B7	NoSites	No
MET_UGT2B15	NoSites	Yes
CYP_Risk	1	0
CYP_Code	mi	
